# IL-31 induces antitumor immunity in breast carcinoma

**DOI:** 10.1136/jitc-2020-001010

**Published:** 2020-08-24

**Authors:** Tal Kan, Erik Feldman, Michael Timaner, Ziv Raviv, Shai Shen-Orr, Ami Aronheim, Yuval Shaked

**Affiliations:** Technion-integrated cancer center, Rappaport Faculty of Medicine, Technion Israel Institute of Technology, Haifa, Israel

**Keywords:** adaptive immunity, cytokines, immunomodulation

## Abstract

**Background:**

Immunomodulatory agents that induce antitumor immunity have great potential for treatment of cancer. We have previously shown that interleukin (IL)-31, a proinflammatory cytokine from the IL-6 family, acts as an antiangiogenic agent. Here, we characterize the immunomodulatory effect of IL-31 in breast cancer.

**Methods:**

In vivo breast carcinoma models including EMT6 and PyMT cell lines were used to analyze the effect of IL-31 on the composition of various immune cells in the tumor microenvironment using high-throughput flow cytometry. In vitro studies using isolated cytotoxic T cells, CD4^+^ T cells, myeloid-derived suppressor cells (MDSCs) and macrophages were carried out to study IL-31 immunological activity. The generation of recombinant IL-31 bound to IgG backbone was used to test IL-31 therapeutic activity.

**Results:**

The growth rate of IL-31-expressing breast carcinomas is decreased in comparison with control tumors due, in part, to antitumor immunomodulation. Specifically, cytotoxic T cell activity is increased, whereas the levels of CD4^+^ T cells, MDSCs, and tumor-associated macrophages are decreased in IL-31-expressing tumors. These cellular changes are accompanied by a cytokine profile associated with antitumor immunity. In vitro, IL-31 directly inhibits CD4^+^ Th0 cell proliferation, and the expression of Th2 canonical factors GATA3 and IL-4. It also promotes CD8^+^ T cell activation through inhibition of MDSC activity and motility. Clinically, in agreement with the mouse data, alterations in immune cell composition in human breast cancer biopsies were found to correlate with high expression of IL-31 receptor A (IL-31Ra). Furthermore, high coexpression of IL-31Ra, IL-2 and IL-4 in tumors correlates with increased survival. Lastly, to study the therapeutic potential of IL-31, a recombinant murine IL-31 molecule was fused to IgG via a linker region (IL-31-L-IgG). This IL-31-L-IgG therapy demonstrates antitumor therapeutic activity in a murine breast carcinoma model.

**Conclusions:**

Our findings demonstrate that IL-31 induces antitumor immunity, highlighting its potential utility as a therapeutic immunomodulatory agent.

## Background

Immunomodulatory agents that promote antitumor immunity have led to major advancements in clinical oncology. Among them are immune checkpoint inhibitors that have demonstrated remarkable success in several advanced malignancies. However, their therapeutic efficacy is limited to a minority of patients.^1^ This has prompted the search for novel immunomodulatory molecules that may be used to improve treatment outcomes in patients who are refractory to immunotherapies or other cancer treatment modalities.^2^

The tumor microenvironment (TME) consists of various cell types that suppress the antitumor immune response. For example, myeloid-derived suppressor cells (MDSCs), T regulatory (Treg) cells, immunosuppressive (M2-like) macrophages,^3–6^ and possible cross-talk between other non-immune cells^7^ all contribute to the suppression of tumor antigen-specific cytotoxic T lymphocytes (CTLs). In addition, CD4^+^ T cells that reside in tumors display Th2 immunity phenotype which has been shown to contribute to tumor growth and poor prognosis in breast cancer as well as in other cancers.^8^ Th2 cells in tumors secrete anti-inflammatory cytokines such as interleukin (IL)-4, IL-13, and IL-10, which inhibit Th1 antitumorigenic immunity and CD8^+^ cytotoxic T cell activity. Moreover, Th2 cytokines further promote the activity of MDSCs, Tregs and M2-like tumor-associated macrophages (TAMs).^9^ TAMs are generally classified into two subtypes, namely M1 and M2 macrophages, with opposing activities. Anti-inflammatory protumorigenic M2-TAMs are associated with a Th2 immune response. They promote tumor growth in part by secreting proangiogenic and immunosuppressive molecules, therefore supporting the immune escape of tumor cells. Conversely, the proinflammatory antitumorigenic M1-TAMs are associated with a Th1 immune response. They exhibit higher major histocompatibility complex II (MHCII) expression, increased phagocytic activity, and express proinflammatory cytokines.^10^ The diverse roles of immune cells in the TME, and the potential to alter their state for therapeutic purposes, are the focus of active research.^8^

We have previously reported that the immunoregulatory cytokine IL-31 acts as an antiangiogenic agent.^11^ IL-31 is a member of the proinflammatory IL-6 cytokine family. It is mainly produced by activated Th2 cells and is associated with atopic dermatitis.^12^ IL-31 acts through a heterodimeric receptor consisting of IL-31 receptor A (IL-31Ra) and oncostatin M receptor, which are coexpressed on various immune cells such as monocytes, dendritic cells, and macrophages,^12 13^ as well as non-immune cells such as keratinocytes and epithelial cells.^14 15^ In IL-31Ra^−/−^ mouse models, it has been shown that IL-31 may negatively regulate type 2 inflammation.^16^ However, the immunomodulatory role of IL-31 in cancer has not been elucidated.

## Methods

### Cell lines and primary cell culture

PyMT murine breast carcinoma cell line was obtained as described in Chang *et al*.^17^ EMT6 murine breast carcinoma and HEK-293T human embryonic kidney cell lines were purchased from ATCC (Manassas, Virginia, USA). Mouse embryonic fibroblast cell line was obtained as described in Weidenfeld-Baranboim *et al*.^18^ Cell lines were cultured in Dulbecco’s modified eagle’s medium (Sigma-Aldrich, Israel) supplemented with 10% fetal bovine serum 1% L-glutamine, 1% sodium pyruvate, and 1% streptomycin-penicillin-neomycin solution (Biological Industries, Israel). All primary cells (lymphocytes, MDSCs, bone marrow cells, macrophages) were cultured in Iscove’s modified Dulbecco’s medium supplemented as described above. Cells were routinely tested to be mycoplasma-free.

### Tumor models

PyMT and EMT6 cells (0.5×10^6^) were implanted into the mammary fat pad of 10-week-old female C57Bl/6 and BALB/c mice, respectively (Envigo, Israel). In some experiments, cells were implanted in 10-week-old female Nonobese Diabetic/Severe Combined Immunodeficiency (NOD-SCID) mice (Envigo, Israel). Tumors were measured with a caliper and volume was calculated according to the formula width^2^ × length × 0.5.

### Single cell preparation from tissue and peripheral blood

Single cell suspensions from tumors or tissue were performed as previously described.^19^ Additional details are provided in the online supplementary material.

10.1136/jitc-2020-001010.supp1 Supplementary data

### Time of flight mass cytometry

BALB/c female mice (10 weeks old) were implanted with mini-osmotic pumps (Alzet, Cupertino, California, USA) containing 400 µg of purified mouse IL-31 (PeproTech, Israel). Control mice underwent the same surgical procedure with empty pumps. After 3 weeks, mice were sacrificed and extracted spleens were prepared as single cell suspensions. The cells were acquired by time of flight mass cytometry (CyTOF) (Fluidigm, South San Francisco, CA, USA) as previously described.^20^ Additional details are provided in the online supplemental material.

### Flow cytometry

Tumor cells, peripheral blood (PB) or spleen suspensions were analyzed by flow cytometry for the different cell populations as indicated in online supplementary table S2. All antibodies were purchased from BioLegend (San Diego, California, USA). Immunostaining was carried out in accordance with the manufacturer’s instructions. In some experiments Ki67-BUV395 (BD Biosciences, San Jose, California, USA) was used. The cells were acquired using LSRFortessa flow cytometer (BD Biosciences). At least 300,000 events were acquired. Data were analyzed using FlowJo V.10 software (FlowJo, Ashland, Oregon, USA).

### In vitro immunological assays

CD4^+^ Th1/Th2 polarization and Th1/Th2 cytokine quantification were performed in accordance with Sekiya and Yoshimura.^21^ MDSC-CD8^+^ T cell co-culture assay was performed as previously described in Timaner *et al*.^22^ Immune cell proliferation assay was carried out using carboxyfluorescein succinimidyl ester proliferation assay (CFSE), as shown in Ten Brinke *et al*.^23^ Bone marrow-derived macrophage (BMDM) isolation and differentiation were carried out as previously shown in Martinez and Gordon and in Trouplin *et al*.^24 25^ The macrophages were then evaluated for phagocytosis activity as shown in Kapellos *et al*.^26^ Additional details are provided in the.

### Lentiviral transduction

To generate stable cell lines overexpressing IL-31 or their counterpart controls, a lentivirus transduction method was used as described in the online supplementary material. Cells transduced with the IL-31-encoding vector are referred to as PyMT-IL-31 and EMT6-IL-31. Control cells transduced with empty vector are referred to as PyMT-ev and EMT6-ev.

### Tumor lysate preparation

Tumor tissue (~50 mg) was placed in a 1.6 mL tube containing 0.1% Triton and protease inhibitor cocktail (1:100; P8340, Sigma-Aldrich, St Louis, Missouri, USA). Stainless steel beads (SSB14B, Next Advance, New York, USA) were added and tumor tissue was homogenized using the Bullet Blender Tissue Homogenizer (Next Advance) according to the manufacturer’s protocol. The homogenate was centrifuged and supernatant was collected. The protein concentration of the tumor lysates was determined using Protein Assay Dye Reagent Concentrate (Bio-Rad, California, USA).

### Enzyme-linked immunosorbent assay

Cell conditioned medium (CM), mouse plasma or tumor lysates were analyzed by specific ELISAs to quantify the levels of IL-31 and Granzyme B (ThermoFisher Scientific, Waltham, Massachusetts, USA, and R&D Systems, Minneapolis, Minnesota, USA, respectively) according to the manufacturers’ instructions.

### Cell viability assay

Control and IL-31-exprssing tumor cells were seeded in a 96-well plate (2000 cells/well). Cell number was evaluated 24 hours and 48 hours later using CellTiter-Glo reagent (Promega, Madison, Wisconsin, USA) according to the manufacturer’s instructions.

### Real-time quantitative PCR

RNA was extracted from cells using Total RNA Purification Kit (Norgen, Ontario, Canada). Complementary DNA (cDNA) was synthesized using High-Capacity cDNA Reverse Transcription Kit (Applied Biosystems, California, USA). Real-time quantitative PCR (RT-qPCR) reaction was performed using SYBR Green Master Mix and run in CFX Connect Real-Time PCR Detection System (Bio-Rad). Analysis was performed using ∆∆Ct method. Primers are listed in online supplementary table S3.

### Transwell motility assay

Spleens from C57bl/6 naïve mice were removed and prepared as single cell suspensions. One million splenocytes were seeded onto 3 µm transwell inserts (BD Biosciences) in serum free media with or without 100 ng/mL IL-31. After 18 hours, the number of cells that had migrated into the lower chamber was evaluated. Specifically, cells from the lower chamber were collected and stained for Gr1-PE (BioLegend). Cell number migrating to the lower chamber was calculated by counting beads (BD Biosciences), in accordance with the manufacturer’s instruction.

### Overall survival correlation analysis

Using the Kaplan-Meier plotter tool^27^ available at https://kmplot.com/analysis/, tumor mRNA levels of IL-31Ra, IL-2, IL-4, and Granzyme B (Affymetrix gene ID 1553032_at, 207849_at, 207539_s_at, and 210164_at, respectively) were correlated with overall survival of patients with breast cancer. Two patient groups were analyzed: the entire group or a subgroup thereof, consisting of patients who received neoadjuvant chemotherapy. Patients were subdivided by trichotomization of lower tercile versus upper tercile. The expression levels of each gene alone or in combination with all other genes were tested. Survival curves were then plotted accordingly. The number of patients for each analysis is listed in the plot.

### Correlation analysis in public data

To evaluate the correlation between the expression of IL-31Ra and IL-4/IL-2, we used a previously collected data set of 104 breast cancer biopsies collected at the time of diagnosis (ie, prior to any treatment with tamoxifen or chemotherapeutic agents) from patients (data available on Gene Expression Omnibus (GEO) as GSE42568). We observed that the mode of the distribution was strongly skewed toward zero, suggesting that in the majority of cases IL-31RA was undetectable. We therefore considered all values 1 SD above the mode as expressed (a total of seven samples listed in online supplementary table S4). These samples were then correlated with IL-4 and IL-2, using average expression of all available probes, as well as with the frequency of cell subsets using the methods described in the following section.

### Cell deconvolution from public data

Cell deconvolution from bulk RNA of human breast tumors was performed as previously described.^28^ Additional details are provided in the online supplementary material.

### IL-31 purification and STAT3-luciferase activation assay

Murine IL-31 cloning and its biological activity testing were performed similar to a previous published study.^11^ Additional details are provided in the online supplementary material.

### Statistical analysis

All in vitro experiments were performed in at least three biological repeats and at least two technical repeats. In the in vivo experiments, mice were randomized, and the number of mice per group is indicated in the figure legends (usually, 5–8 mice/group). The data are presented as mean±SD. Statistical significance between two groups was evaluated by two-tailed Student’s t-test. When data sets were not distributed in a Gaussian manner, statistical significance was evaluated with a non-parametric Mann-Whitney test. When comparing more than two groups, statistically significant differences were assessed by one-way analysis of variance, followed by Tukey post-hoc test. Analysis was carried out using GraphPad Prism V.4 software (La Jolla, California, USA). Significance was set at values of p<0.05.

## Results

### IL-31 induces local and systemic antitumor immunity

**Figure 1:**
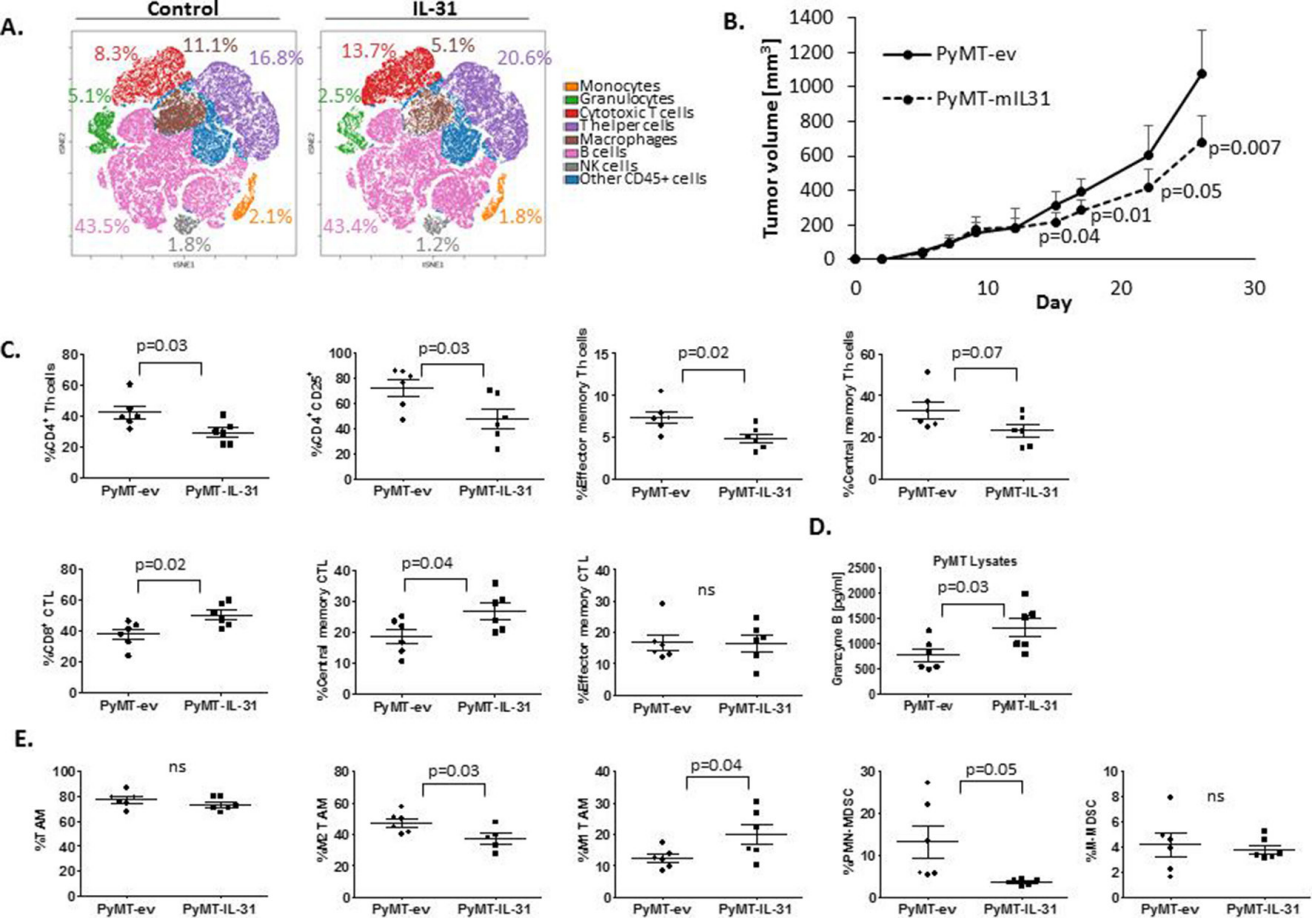
IL-31 expression in tumors enhances antitumor immunity. (A) Non-tumor-bearing mice were infused with IL-31 via osmotic pumps (~14 µg/day) or control (n=5 mice/group). Three weeks later, mice were sacrificed and spleens were removed. Splenocytes (pooled per group) were immunostained with a 37-antibody panel as described in online supplementary table S1. Cells were acquired by CyTOF and data were analyzed by Cytobank. The analysis of the different immune cell populations found in the spleens is presented by viSNE plot. (B–D) PyMT cells stably overexpressing IL-31 (PyMT-IL-31) or their counterpart control cells (PyMT-ev) were implanted in the mammary fat pads of 10-week-old C57BL/6 female mice (n=6 mice/group). Tumor volume was measured and plotted (B). At endpoint, tumors were removed and prepared as single cell suspensions. Lymphoid cells (C), Granzyme B expression in tumor lysates (D), and myeloid cells (E) were assessed. Statistical significance was assessed by unpaired two-tailed t-test. Significant p values are shown. CTL, cytotoxic T lymphocyte; CyTOF, time of flight mass cytometry; IL-31, interleukin-31; MDSCs, myeloid-derived suppressor cells; NK, natural killer; ns, non-significant; PMN, polymorphonuclear; TAM, tumor-associated macrophage; Th, T helper.

To characterize the immunomodulatory role of IL-31, we first analyzed the changes in various immune cell populations on IL-31 infusion in tumor-free mice. To this end, BALB/c mice were implanted with Alzet osmotic minipumps that infused ~14 µg of recombinant IL-31 per day, for 3 weeks. Subsequently, mice were sacrificed and PB and spleens were harvested. Single cell suspensions were analyzed by mass cytometry (CyTOF) and Cytobank online software. In comparison with control mice, spleens of IL-31-treated mice exhibited an increase in the frequency of CTL (CD8^+^) and T helper (CD4^+^) cells and a reduction in the frequency of polymorphonuclear (PMN) MDSCs (Ly6C^dim^Ly6G^+^) as assessed by the viSNE plot (figure 1A). In particular, among the T cell population, central memory cells (CD62L^+^CD44^+^) were increased (online supplementary figure S1A). In contrast, the macrophage population (CD11b^+^F4/80^+^) was decreased in the spleens of IL-31-treated mice and skewed toward the M1-like state, as evaluated by decreased expression of the M2 marker CD206 and increased expression of CD80, CD86 and MHC class II (IA/IE) markers (online supplementary figure S1B). In addition, the levels of CD11b^+^Gr1^+^ and CD4^+^CD25^+^ cells, which represent MDSCs and a subpopulation of T cells, respectively, were decreased in the PB and spleens of IL-31-treated mice (online supplementary figure S1C). In comparison with control mice, no differences were found in the frequency of NK cell population (figure 1A). Collectively, these results indicate that IL-31 treatment promotes a more proinflammatory state in both lymphoid and myeloid lineages.

To create a model for investigating whether the immunomodulatory function of IL-31 affects tumor progression, we generated stable IL-31-expressing PyMT and EMT6 breast carcinoma cell lines (PyMT-IL-31 and EMT6-IL-31, respectively) using lentiviral transduction. The PyMT breast carcinoma model is used to study Luminal B breast carcinoma,^29^ whereas EMT6 model is considered to be triple negative breast carcinoma.^30^ Control cells were stably transduced with empty vector to generate PyMT-ev and EMT6-ev cell lines. Both IL-31-expressing cell lines secreted high levels of IL-31 and exhibited similar proliferation rates in comparison with their counterpart controls (online supplementary figure S2A). To investigate the effect of IL-31 expression on tumor growth, mice were orthotopically implanted with PyMT-IL-31, EMT6-IL-31 or respective control cells. Tumor growth rates were decreased in mice implanted with PyMT-IL-31 or EMT6-IL-31 cells, in comparison with controls. Notably, the delay in tumor growth was more pronounced in the PyMT model (figure 1B and online supplementary figure S2B). High circulating plasma and tumor levels of IL-31 were confirmed in both models (online supplementary figure S2C), and in the PyMT model a significant inverse correlation was found between IL-31 plasma levels and tumor size (online supplementary figure S2D). We also found that mice implanted with PyMT-IL-31 cells developed alopecia over time (online supplementary figure S2E), as was previously reported in IL-31-overexpressing mice^12^; however, other expected effects, for example, pruritus, were not specifically noticed in our mice. At endpoint, immune cell populations in tumors and PB were analyzed. PyMT-IL-31 tumors exhibited significant changes in the levels of various immune cells in comparison with control PyMT-ev tumors. Specifically, the level of total CD4^+^ T cells was decreased, while the level of CD8^+^ T cells was increased (figure 1C). Accordingly, elevated levels of Granzyme B in PyMT-IL-31 tumor model indicate enhanced cytotoxicity (figure 1D). Immunophenotyping of the tumor-infiltrating lymphocyte populations revealed a decrease in the levels of CD4^+^CD25^+^ T cells and CD4^+^ effector memory cells and an increase in the population of central memory CD8^+^ T cells (figure 1C). Since CD4^+^CD25^+^ may represent a subset of Treg cells, we analyzed CD4^+^CD25^+^ cells expressing the transcription factor Forkhead Box P3 (FOXP3), therefore suggesting that they are Tregs. No changes in the levels of Tregs were observed in PyMT-ev and PyMT-IL-31 tumors (online supplementary figure S2F). Focusing on the myeloid lineage, the levels of immunosuppressive M2 macrophages and PMN-MDSCs were decreased, whereas the proinflammatory M1 macrophage population was increased (figure 1E). In PB of mice bearing PyMT-IL-31 tumors, the levels of CD4^+^CD25^+^ T cells and PMN-MDSCs were slightly but significantly decreased (online supplementary figure S2G), in accordance with the changes detected in PyMT-IL-31 tumors. In the EMT6-IL-31 tumors, an increase in CD8^+^ and decrease in CD4^+^ T cell frequencies were notable in comparison with control (online supplementary figure S2H); however, no significant changes were detected in the levels of myeloid immune populations (online supplementary figure S2I) nor of Granzyme B (online supplementary figure S2J). This may explain the weaker antitumor effect observed in the EMT6 tumor model.

Next, to evaluate whether the antitumor activity of IL-31 is associated with changes in systemic immune cells, we implanted PyMT-ev or PyMT-IL-31 cells in the mammary fat pad of naïve mice, while in the opposing mammary fat pad we implanted wildtype PyMT cells (referred to as PyMT-wt(ev) and PyMT-wt(IL-31), respectively), as illustrated in online supplementary figure S3A. In accordance with the data above, PyMT-wt tumors that were paired with IL-31-secreting tumors exhibited a decreased growth rate in comparison with PyMT-wt tumors that were paired with control tumors (online supplementary figure S3B). We next focused on the immune cell phenotype in PyMT-wt(ev) versus PyMT-wt(IL-31) tumors. In compliance with our previous results, a significant increase in CTL frequency and increase in Granzyme B levels were found in PyMT-wt(IL-31) tumors compared with PyMT-wt(ev) (online supplementary figure S3C, D). Moreover, in these tumors there was a significant inhibition of CD4^+^ Th cells as suggested by a decreased frequency of CD4^+^CD25^+^ T cells, effector memory and central memory Th cell populations. Likewise, suppressive myeloid populations such as M2-TAMs and Ly6C^dim^Ly6G^+^ PMN-MDSCs were decreased as well (online supplementary figure S3C). We confirmed that IL-31 levels were increased only in tumor lysates of PyMT-IL-31 primary tumor and not in PyMT-ev, PyMT-wt(ev) and PyMT-wt(IL-31) as well as in the plasma of PyMT-IL-31 tumor-bearing mice (online supplementary figure S3E). Finally, to confirm that the antitumorigenic effect mediated by IL-31 is highly dependent on immune cells, we implanted PyMT-ev and PyMT-IL-31 cells into the mammary fat pad of immune-deficient female NOD-SCID mice. These mice lack both myeloid and lymphoid immune system.^31^ Tumor growth did not differ between PyMT-IL-31 and PyMT-ev tumors (online supplementary figure S3F). Taken together, IL-31 induces systemic antitumor immunity by diminishing immunosuppressive cell populations and increasing CTL populations in tumors and the circulation.

### IL-31 inhibits the expression of Th2-related cytokines in peripheral Th cells and in the TME

**Figure 2:**
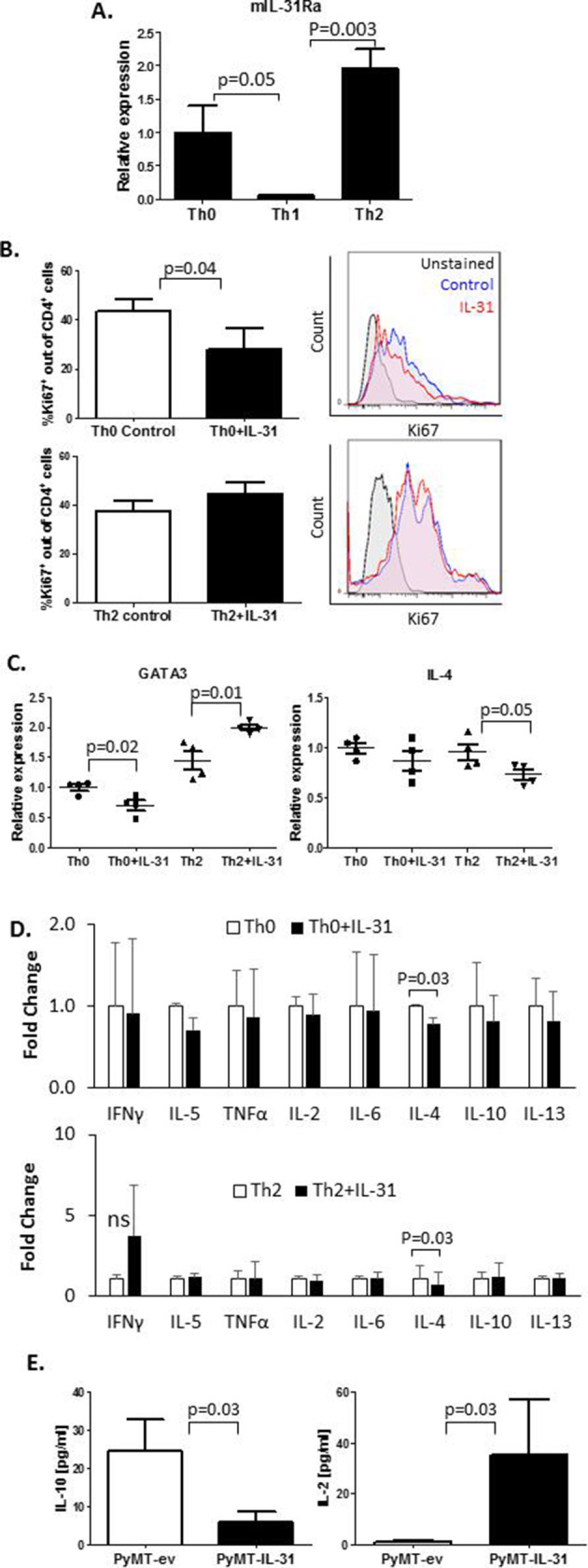
IL-31 affects CD4^+^ T cell proliferation and cytokine secretion. (A–D) CD4^+^ T cells were isolated from spleens of naïve C57BL/6 female mice by immunomagnetic negative selection. The cells were left untreated (Th0) or skewed toward Th1 or Th2, as described in the Methods section. IL-31Ra mRNA expression was evaluated by RT-qPCR (n=3 biological repeats, A). CD4^+^ T cells were activated with anti-CD3 and anti-CD28 to generate Th0 cells or cultured in skewing conditions to generate Th2 cells. Th0 and Th2 cells were exposed to 100 ng/mL IL-31 or left untreated for 4 days. Cells were fixed and immunostained with Ki67 to evaluate proliferation rate (n=3–4 biological repeats). Representative Ki67 histograms and summary quantification graphs are shown (B). GATA3 and IL-4 mRNA levels were measured by RT-qPCR; n=4 biological repeats (C). The levels of various Th1/Th2 cytokines were quantified in CM of control and IL-31-treated Th0 and Th2 cultures. Fold changes relative to control are shown (D). (E) IL-10 and IL-2 cytokine concentrations in PyMT-ev and PyMT-IL-31 tumor lysates were evaluated (n=4–6 mice/group). Statistical significance was assessed by one-way analysis of variance, followed by Tukey post-hoc test (A, C), unpaired two-tailed t-test (B, E), or paired two-tailed t-test (D). Significant p values are shown. CM, cell conditioned medium; IFNγ, interferon-γ; IL, interleukin; IL-31Ra, IL-31 receptor A; ns, non-significant; RT-qPCR, real-time quantitative PCR; Th, T helper; TNF-α, tumor necrosis factor-α.

Th2 cells highly infiltrate breast carcinomas and contribute to the immunosuppressive environment of the tumor milieu.^32^ Moreover, it has been reported that IL-31 is involved in type 2 responses.^12^ We therefore sought to explore the effect of IL-31 on Th1 and Th2 immunity in tumors. Since IL-31 systemically contributes to antitumor immunity (online supplementary figure S3), we asked whether there is a direct effect between IL-31 and peripheral CD4^+^ T cells. Using RT-qPCR we analyzed IL-31Ra expression in CD4^+^ T cells isolated from spleens of naïve mice. CD4^+^ cell isolation reached over 90% purity (online supplementary figure S4A). The cells that were skewed to Th2 or left as Th0 demonstrated significantly higher mRNA levels of IL-31Ra in comparison with those that were skewed toward Th1 (figure 2A). We found that IL-31 decreased the cell proliferation rate of Th0 but not Th2 cells (figure 2B). Furthermore, IL-31 significantly decreased mRNA expression of the Th2 master regulator GATA binding protein 3 (GATA3) in Th0 cells while increasing its expression in Th2 cells (figure 2C). Interestingly, IL-4 mRNA levels were decreased in IL-31-treated Th2 cells, although GATA3 levels were elevated. However, no changes in IL-4 mRNA levels were found in Th0 (figure 2C). Nevertheless, the addition of IL-31 to cultured cells resulted in decreased soluble IL-4 levels in both Th0 and Th2 cell CM (figure 2D). The presence of IL-31 did not significantly change mRNA levels of the Th1 inducers, T-bet and interferon-γ (IFN-γ) (online supplementary figure S4B) or other Th1/Th2 soluble cytokines secreted by Th0 and Th2 cells (figure 2D), further indicating that IL-31 negatively regulates Th2 polarization without altering Th1 canonical factors.

To determine the net effect of IL-31 on the immune state in the TME, we evaluated the levels of a repertoire of Th1/Th2 cytokines in whole tumor lysates of PyMT-ev and PyMT-IL-31 tumors. A significant decrease in IL-10 and a significant increase in IL-2 were observed in PyMT-IL-31 when compared with PyMT-ev tumors (figure 2E). The levels of other tested Th1/Th2 cytokines did not significantly change between the two groups (online supplementary figure S4C). Taken together, these results suggest that IL-31 promotes antitumor immunity by suppressing Th2-related cytokines in peripheral Th cells and in the TME.

### IL-31 promotes cytotoxic T cell frequency and inhibits MDSC motility

As CD8^+^ T cell subpopulations are increased in IL-31-expressing tumors (figure 1C), we asked whether IL-31 induces a direct effect on these cells. To this end, we isolated CD8^+^ cells from the spleens of non-tumor-bearing C57bl/6 mice, reaching purity of over 95% (online supplementary figure S5A), and assessed whether IL-31 affects their activation or proliferation in vitro. IL-31 had no apparent effect on the activation state or proliferation rate of the isolated splenic CD8^+^ T cells (online supplementary figure S5B), suggesting that it acts indirectly.

**Figure 3:**
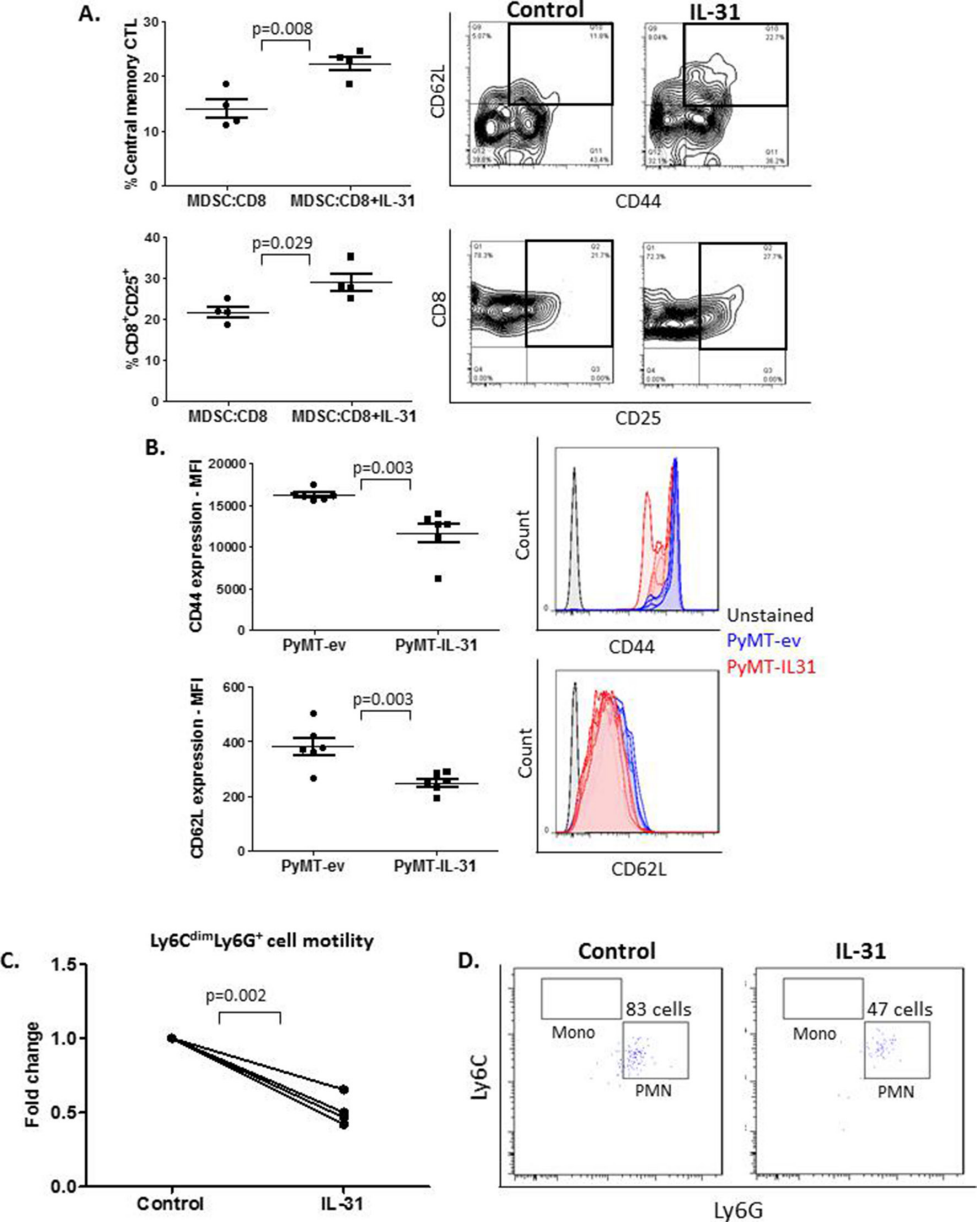
IL-31 promotes a cytotoxic T cell memory phenotype and inhibits MDSC motility. (A) CD8^+^ T cells isolated from naïve mice were co-cultured with Gr1^+^ MDSCs isolated from PyMT tumors. T cells were activated with soluble anti-CD3 and anti-CD28. Cultures were then supplemented with 100 ng/mL IL-31 or left untreated. After 4 days, the activation and memory phenotype of CD8^+^ T cells were evaluated by flow cytometry. (B) The expression of CD44 and CD62L was evaluated in PMN-MDSCs isolated from peripheral blood of mice implanted with PyMT-ev or PyMT-IL-31 tumors. The evaluation was performed by flow cytometry using MFI. (C, D) The motility of MDSCs was measured by seeding one million splenocytes from naïve C57BL/6 mice (n=4 biological repeats) on a 3 µm insert containing SF media in both upper and lower compartments. The cells were treated with 100 ng/mL IL-31 or left untreated (control). After 18 hours, the cells in the lower compartment were collected, stained for Ly6G and Ly6C, and counted by flow cytometry. A summary graph is shown (C) followed by representative flow cytometry dot-plots (D). Statistical significance was assessed by unpaired two-tailed t-test. Statistical significance of MDSC motility was assessed by paired t-test. Significant p values are shown. CTL, cytotoxic T lymphocytes; IL-31, interleukin-31; MDSCs, myeloid-derived suppressor cells; MFI, mean fluorescence intensity; PMN, polymorphonuclear; SF, serum free media.

Next, MDSCs are known to regulate CTL activity and expansion.^33^ Since we demonstrated that the frequency and activity of CD8^+^ CTLs were increased in PyMT-IL-31 tumors (figure 1C), we hypothesized that IL-31 regulates immunosuppressive MDSCs, which in turn may reduce their ability to suppress CD8^+^ T cells. To test our hypothesis, Gr1^+^ MDSCs (both monocytic and PMN-MDSCs) were isolated from PyMT tumors (purity >95%; online supplementary figure S5C) and co-cultured in a 1:1 ratio with splenic CD8^+^ cells isolated from naïve mice. CD8^+^ T cells were activated using anti-CD3/CD28 and cultured in the presence or absence of IL-31. Four days later, the levels of various T cell subpopulations were evaluated. Indeed, IL-31 significantly increased the frequency of CD8^+^ T cells with central memory phenotype (CD62L^+^CD44^+^) in the co-culture system (figure 3A). Moreover, CD25^+^ CTL frequency was significantly elevated without an apparent change in proliferation rate (figure 3A and online supplementary figure S5D). Of note, the frequency of effector memory CTLs and the levels of various anti-inflammatory and proinflammatory cytokines did not differ between the two groups (online supplementary figure S5E, F).

Next, since MDSC frequency was decreased in PyMT-IL-31 tumors and PB of mice implanted with PyMT-IL-31 (figure 1E), we asked whether IL-31 affects the adhesive and migratory properties of MDSCs, thereby explaining their reduced infiltration to tumors. In vivo, we found that almost all PB PMN-MDSCs were positive for the adhesion molecules CD62L and CD44 in both PyMT-ev and PyMT-IL-31 groups (online supplementary figure S5G). However, the expression levels of these molecules were significantly reduced in PMN-MDSCs from PyMT-IL-31 tumor-bearing mice in comparison with those from PyMT-ev tumor-bearing mice (figure 3B). Furthermore, we found that the addition of IL-31 to cultures of whole splenocytes resulted in inhibition of Ly6C^dim^Ly6G^+^ PMN cell motility in comparison with untreated cells (figure 3C, D). Of note, Ly6C^hi^Ly6G^−^ monocytic MDSCs did not show any motility capability in our experimental setting. These results suggest that IL-31 inhibits PMN-MDSC motility which might decrease their ability to infiltrate tumors. Overall, these results suggest that IL-31 promotes CTL activation by reducing the presence of PMN-MDSCs in tumors.

### IL-31 induces a macrophage proinflammatory function

**Figure 4:**
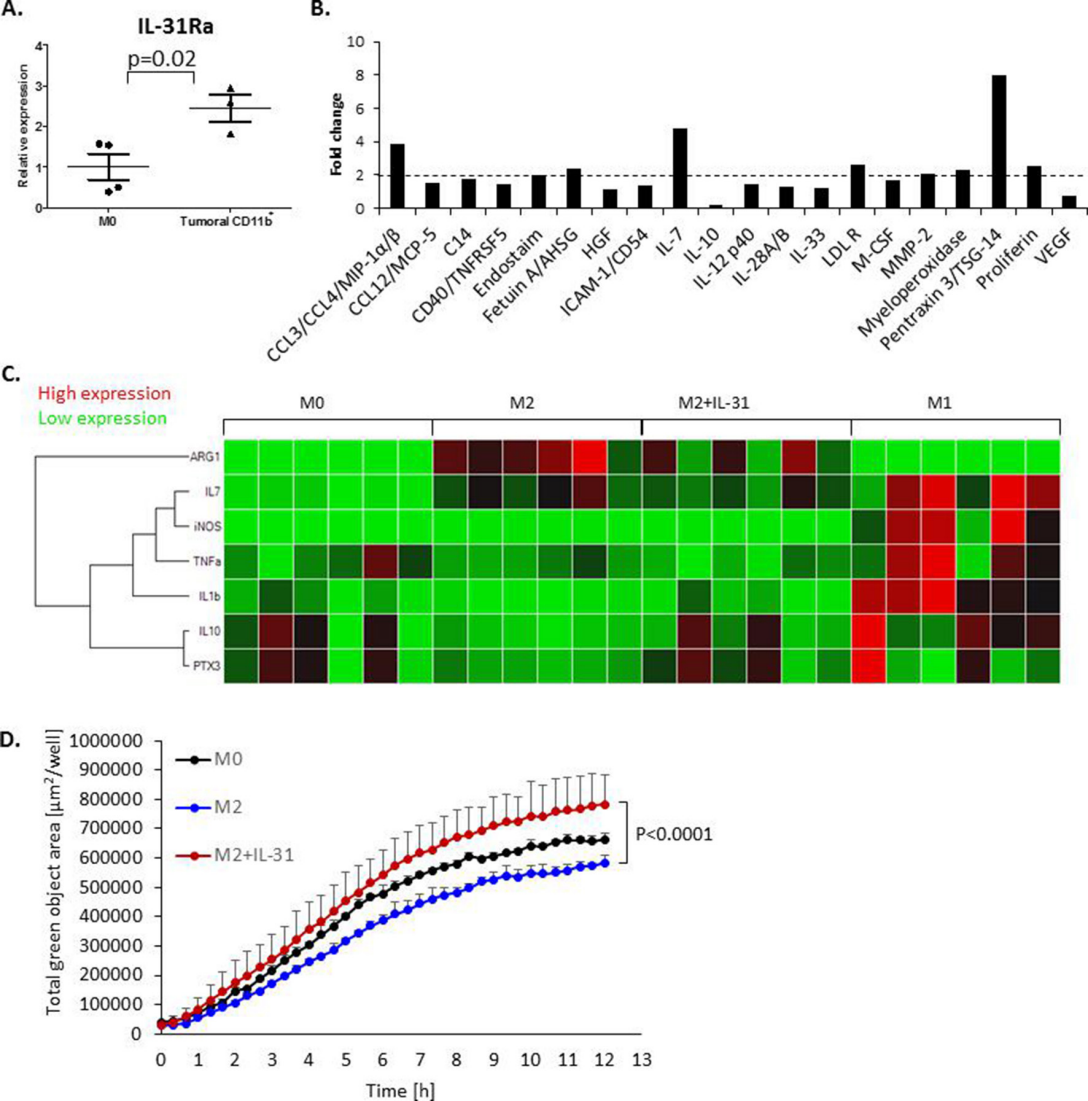
IL-31 induces a proinflammatory state in macrophages and enhances macrophage phagocytic activity. (A) The level of IL-31Ra mRNA was measured by RT-qPCR in naïve M0 BMDMs and in CD11b^+^ myeloid cells isolated from PyMT tumors (n=3–4 biological repeats). (B, C) BMDMs were isolated from naïve female C57BL/6 mice and skewed toward the M2 phenotype (by IL-4 treatment), the M1 phenotype (by interferon-γ treatment) or left as the M0 phenotype (no additives). M2 BMDMs were treated with 100 ng/mL IL-31 or left untreated (control). The levels of various secreted proteins in the conditioned medium were measured. Data are presented as fold change relative to control. Dashed line represents all proteins which displayed over twofold increase (B). The mRNA expression levels of various inflammatory and anti-inflammatory factors were evaluated in untreated M0, M1 and M2 BMDMs, and IL-31-treated M2 BMDMs (M2+IL-31). Shown is a heat map of normalized values (C). (D) The phagocytic activity of untreated M0, untreated M2 and IL-31-treated M2 macrophages (M2+IL-31) was measured by IncuCyte. Statistical significance was assessed by unpaired two-tailed t-test. Significant p values are shown. BMDMs, bone marrow-derived macrophages; IL, interleukin; IL-31Ra, IL-31 receptor A; RT-qPCR, real-time quantitative PCR; AHSG, alpha 2-heremans schmid glycoprotein; ICAM-1, intercellular adhesion molecule 1; HGF. hepatopoietin-A; LDL R, low density lipoprotein receptor; M-CSF, macrophage colony-stimulating factor; MMP-2, matrix metalloproteinase-2; TSG-14, tumor necrosis factor-inducible gene 14; VEGF, vascular endothelial growth factor; iNOS, inducible nitric oxide synthase; TNF-a, tumor necrosis factor alpha; PTX3, pentraxin 3.

We have shown that IL-31 affects the activation and M1/M2 polarization of macrophages from spleens of non-tumor-bearing mice infused with IL-31 as well as from PyMT-IL-31 tumors (figure 1E and online supplementary figure S1B). These results prompted us to further elucidate the direct effect of IL-31 on macrophages. IL-31Ra mRNA expression was increased in myeloid cells from PyMT tumors in comparison with naïve macrophages (figure 4A), implicating that IL-31 may directly affect macrophages. Next, BMDMs were generated and confirmed for their phenotype and purity (online supplementary figure S6A). In line with a previous study, IL-31Ra expression was induced in M2-like BMDMs (online supplementary figure S6B).^34^ Yet no differences were found in the expression of CD11c and CD206, surface markers of macrophages, when M2 macrophages were cultured in the presence or absence of IL-31 (online supplementary figure S6C). Thus, IL-31 does not directly affect the phenotype of macrophages in our in vitro setting.

Next, we further evaluated the functional properties of macrophages by analyzing their secretion profiles in vitro in the presence or absence of IL-31. IL-31-treated M2 macrophages secreted higher levels of various factors associated with recruitment, activation, and survival of lymphocytes such as MIP1a/b and IL-7 and lower levels of IL-10, in comparison with untreated control M2 macrophages. Moreover, IL-31-treated macrophages secreted higher levels of Pentraxin 3 (PTX3), a protein involved in inhibition of metastasis and contributing to macrophage phagocytosis activity^35 36^ (figure 4B). The expression patterns of these and additional proinflammatory and anti-inflammatory factors were evaluated in IL-31-treated M2 macrophages, comparing with expression patterns in M0 untreated macrophages, IL-4-exposed M2 macrophages and lipopolysaccharide (LPS)-exposed M1 macrophages. Specifically, in the IL-31-treated M2 macrophages, the expression pattern of M1-like and M2-like cytokines resembled an intermediate state between M2 and LPS-treated M1 macrophages (figure 4C). These results suggest that IL-31 induces macrophage skewing toward a proinflammatory functional state.

To further verify that macrophage activity is altered in response to IL-31, we evaluated the phagocytic function of M2 macrophages in the presence of IL-31. To this end, BMDMs were cultured with Green Zymosan Bioparticles and a time course analysis was performed. IL-31 increased the phagocytic activity of M2 macrophages in comparison with untreated M0 macrophages or IL-4-treated M2 macrophages (figure 4D). Overall, our results suggest that even though IL-31 did not induce an M2 to M1 phenotypic change in macrophages, it did promote functional skewing toward a proinflammatory state. Thus, IL-31 directly modulates macrophage activity and protein secretion toward a proinflammatory state, which can explain the antitumorigenic phenotype in the tumor.

### High coexpression of IL-31Ra, IL-2 and IL-4 in tumors correlates with overall survival in patients with breast cancer

**Figure 5:**
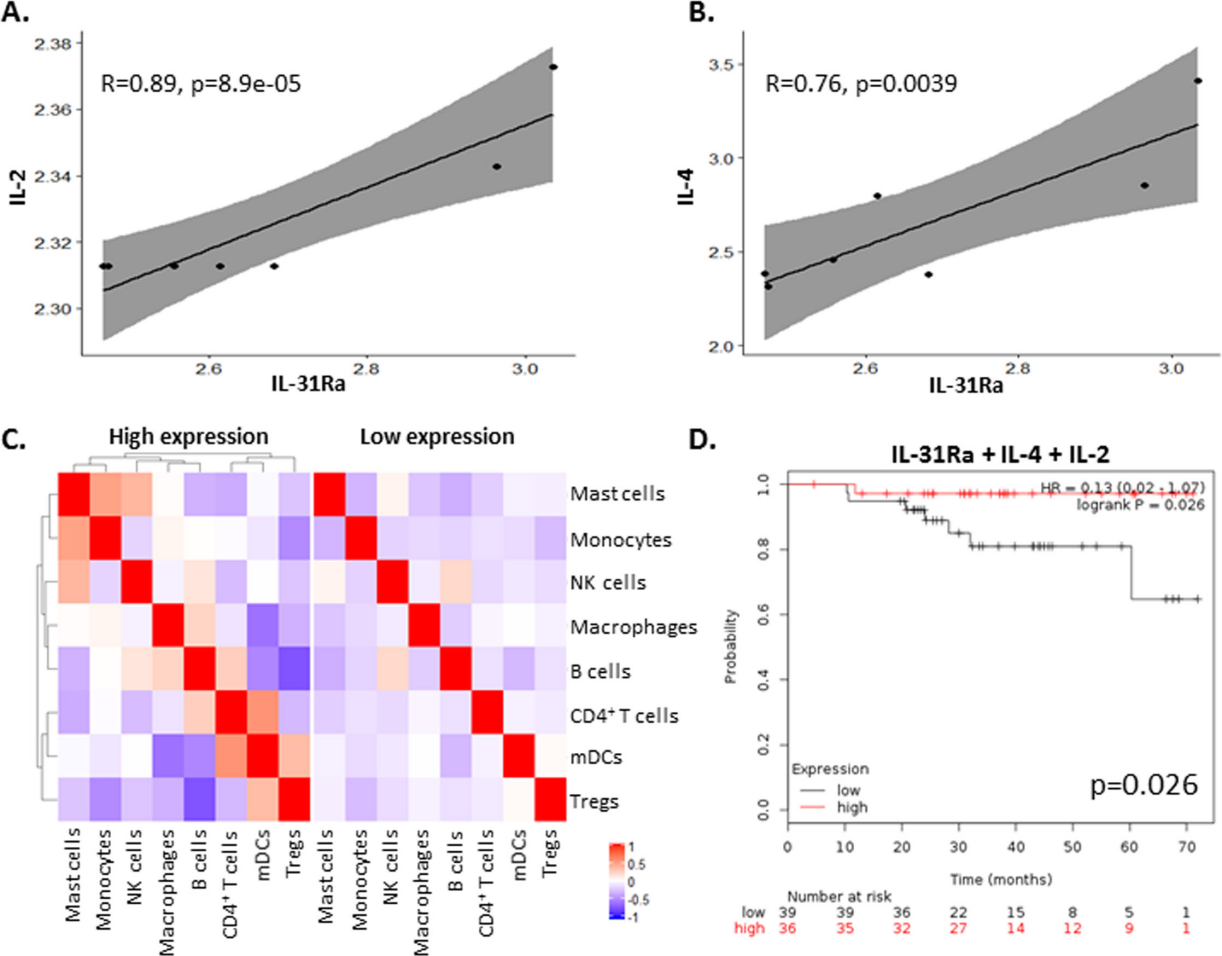
High IL-31Ra expression in breast tumors from patients correlates with IL-2 and IL-4 and contributes to immune modulation and overall survival. (A, B) Pearson correlation analysis was performed on mRNA levels of IL-31Ra relative to IL-2 (A) or IL-4 (B) mRNA levels from Affymetrix data sets of patients with breast cancer. (C) Cell deconvolution was performed on bulk mRNA data set of patients with breast cancer to identify immune subsets present in the tumor microenvironment. The correlation between the different immune cell subsets and IL-31Ra expression was plotted and compared with patients where IL-31Ra expression was not detected. Red indicates positive correlation. Blue indicates negative correlation. (D) Kaplan-Meier curves of patients with breast cancer who received neoadjuvant chemotherapy were plotted as a function of mRNA levels of IL-31Ra, IL-2, and IL-4. IL, interleukin; IL-31Ra, IL-31 receptor A; Treg, T regulatory cells; NK, natural killer; mDCs, myeloid dendritic cells.

Since PyMT tumors that overexpress IL-31 exhibit an increase in IL-2 concentrations (figure 2E), we investigated whether there is a correlation between its expression and IL-31 or IL-31Ra in breast carcinoma biopsies from patients. To this end, we analyzed independent Affymetrix data sets of mRNA expression levels from breast cancer tissues using the Kaplan-Meier plotter available online as described in the Methods section. In agreement with our findings in mice, a significant positive correlation between IL-31Ra and IL-2 mRNA levels was found in human breast tumors (figure 5A). Of note, since IL-4 is known to induce the expression of IL-31Ra,^34^ we also observed a significant positive correlation between IL-31Ra and IL-4 mRNA levels (figure 5B). No significant correlations were found between IL-31Ra and IL-10 or Granzyme B expression levels (data not shown). Next, to dissect the effect of IL-31Ra on immune cell composition in tumors from patients with breast cancer, we assessed the different immune cell populations by bulk mRNA from tumor biopsies using cell deconvolution method.^28^ Patients’ characteristics are listed in online supplementary table S4. Only in IL-31Ra-expressing tumors there were alterations in immune cell composition and correlation between different immune cell subsets when compared with tumors with undetectable mRNA levels of IL-31Ra (figure 5C), indirectly suggesting that the IL-31-IL-31Ra axis contributes to immune modulation. Finally, to determine whether expression of IL-31 or IL-31Ra correlates with patient survival, we generated survival curves from independent data sets using the Kaplan-Meier plotter tool as previously published.^27^ We first focused only on patients who were treated with neoadjuvant chemotherapy. IL-31Ra mRNA expression did not correlate with increased survival (online supplementary figure S7A). However, we found that high coexpression of IL-31Ra, IL-4 and IL-2 was associated with increased overall survival (figure 5D). Notably, there was no correlation between survival and the expression of IL-4 and/or IL-2 (online supplementary figure S7A).

Next, focusing on data from the entire cohort of patients with breast cancer, we found that high coexpression of IL-31Ra, IL-4, IL-2, and Granzyme B was associated with increased survival, with results almost reaching statistical significance (p=0.056; online supplementary figure S7B). Importantly, there was no correlation with survival if IL-31Ra was excluded from the list (online supplementary figure S7C). These results suggest that IL-31/IL-31Ra signaling may contribute to increased survival in patients with breast cancer due to modulation of antitumor cytokines and other immune components which support antitumor activity.

### IL-31 has an immunomodulatory therapeutic effect in a murine breast carcinoma model

In light of our findings that demonstrate the role of IL-31 in antitumor immunity, we evaluated the potential of using IL-31 as a therapeutic intervention for cancer, similar to other immunomodulatory cytokines such as IL-2.^37^ A significant proportion of cytokines has a short half-life in the circulation.^38^ Previously, we designed a recombinant human IL-31 cytokine fused to an IgG backbone that was stable in the blood and demonstrated antitumor activity in vivo.^11^ However, a similarly designed murine IL-31-IgG was unable to induce IL-31Ra signaling in vitro. Since human and murine IL-31 share only 31% homology,^12^ there might be a possible allosteric interference from the IgG backbone that affects the binding of IL-31 to its receptor. To overcome this possibility, we designed a new murine IL-31-IgG molecule in which a 10-amino acid linker was inserted between IL-31 and IgG (designated as IL-31-L-IgG; online supplementary figure S8A). Indeed, IL-31-L-IgG demonstrated significant activity at a dose of 5 µg/mL in vitro (online supplementary figure S8B).

**Figure 6:**
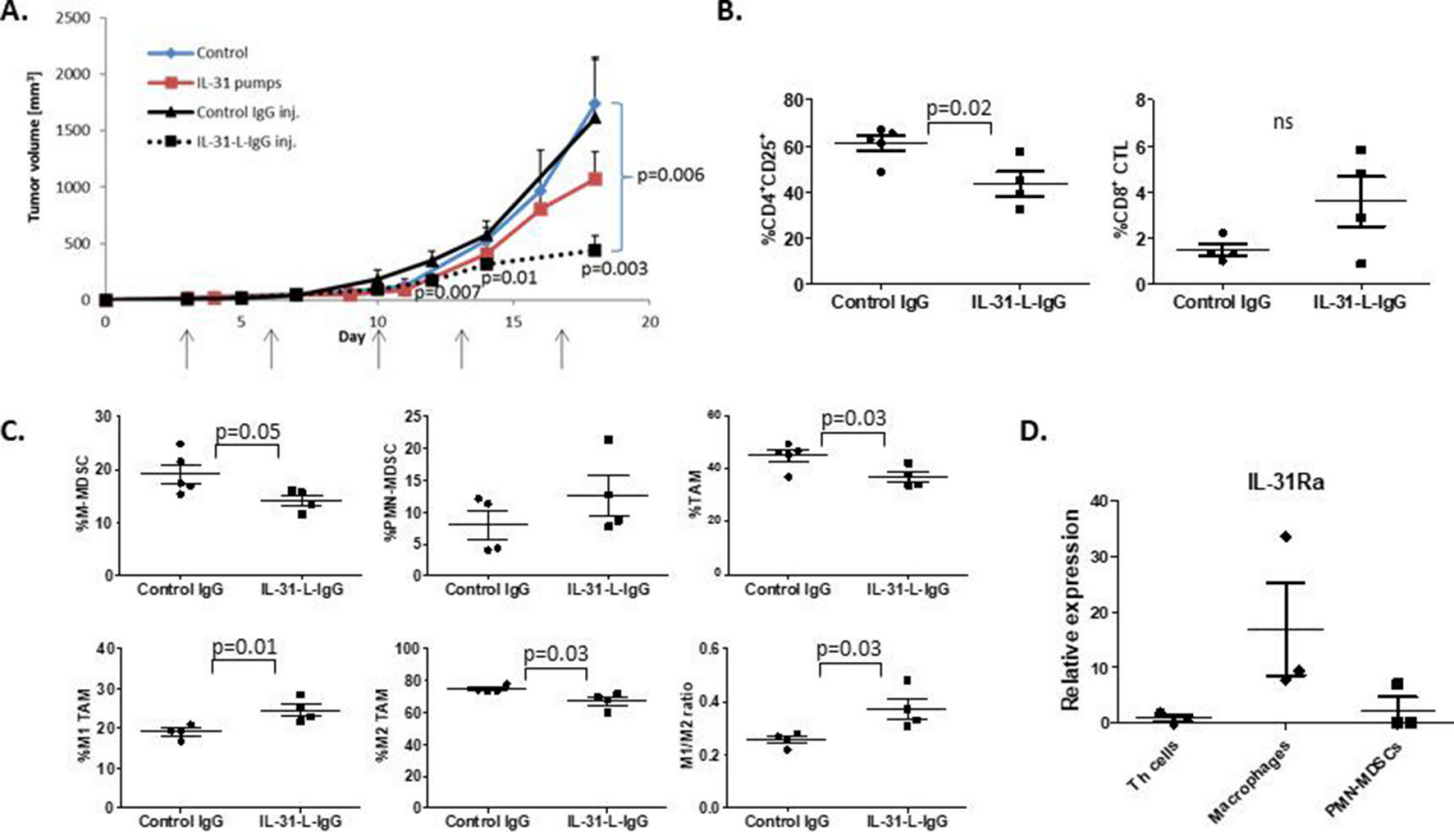
IL-31 displays potent antitumor activity in vivo. Ten-week-old C57BL/6 female mice were implanted with PyMT cells in the mammary fat pad. After 3 days, mice were intraperitoneally injected with IL-31-L-IgG or control IgG twice weekly (100 µg/inj.). In parallel, another group was implanted with mini-osmotic pumps that delivered purified IL-31 at a dose of 17 µg/day. Control mice were implanted with empty pumps (n=4–5 mice/group). (A) PyMT tumor growth was measured biweekly. (B, C) At endpoint, tumors from mice treated with IL-31-L-IgG or IgG were removed and prepared as single cells. Lymphoid (B) and myeloid (C) populations were analyzed by flow cytometry. (D) Th (CD4^+^) cells, macrophages (CD11b^+^F4/80^+^), and PMN-MDSCs (Ly6C^dim^Ly6G^+^) were isolated from the spleen of PyMT tumor-bearing C57Bl/6 mice (n=3). RNA was extracted and IL-31Ra mRNA expression was analyzed by RT-qPCR. Relative mRNA levels are present. Statistical significance was assessed by one-way analysis of variance, followed by Tukey post-hoc test for (A) and (D), or by unpaired two-tailed t-test for (B) and (C). Significant p values are shown. CTLs, cytotoxic T lymphocytes; IL, interleukin; IL-31Ra, IL-31 receptor A; MDSCs, myeloid-derived suppressor cells; ns, non-significant; PMN, polymorphonuclear; RT-qPCR, real-time quantitative PCR; TAM, tumor-associated macrophages; Th, T helper.

We next analyzed the therapeutic potential of IL-31-L-IgG in a mouse model of breast carcinoma. To this end, C57Bl/6 mice were orthotopically implanted with PyMT murine breast carcinoma cells. After 3 days, mice were intraperitoneally injected with 100 µg IL-31-L-IgG or control IgG twice a week. As a positive control, mice were implanted with osmotic minipumps containing 500 µg purified IL-31. Both IL-31-L-IgG and purified IL-31 inhibited tumor growth in comparison with controls. Notably, the antitumor effect of IL-31-L-IgG was more potent than that of purified IL-31 (figure 6A). At endpoint, mice were sacrificed, blood was drawn, and tumors were harvested. Immune cells in PB and tumors were analyzed by flow cytometry. Tumors from mice treated with IL-31-L-IgG contained significantly lower levels of CD4^+^CD25^+^ T cells, TAMs and MDSCs in comparison with tumors from control IgG-treated mice. The level of CD8 cytotoxic T cells was elevated in tumors from IL-31-L-IgG-treated mice, although data were not statistically significant (figure 6B, C). In PB, populations of CD4^+^CD25^+^ T cells and MDSCs were significantly decreased in IL-31-L-IgG-treated mice (online supplementary figure S8C). Of note, Treg population in the tumor did not differ between the groups (online supplementary figure S8D). These effects are in agreement with those observed in PyMT tumors in which IL-31 is overexpressed (figure 1).

We next asked which of the immunomodulatory effects of IL-31 on different immune cells is most pronounced. To address this, we isolated CD4^+^ cells, macrophages, and PMN-MDSCs from the spleen of PyMT tumor-bearing mice and analyzed the expression mRNA levels of IL-31Ra. Our analysis revealed that IL-31Ra is highly expressed in macrophages compared with all other cells, indicating that IL-31 predominant effect is likely to be on macrophages (figure 6D). Overall, our results demonstrate that IL-31 induces antitumor immunity by affecting various immune cells in a breast cancer model, highlighting the potential use of IL-31 as a therapeutic intervention.

## Discussion

**Figure 7:**
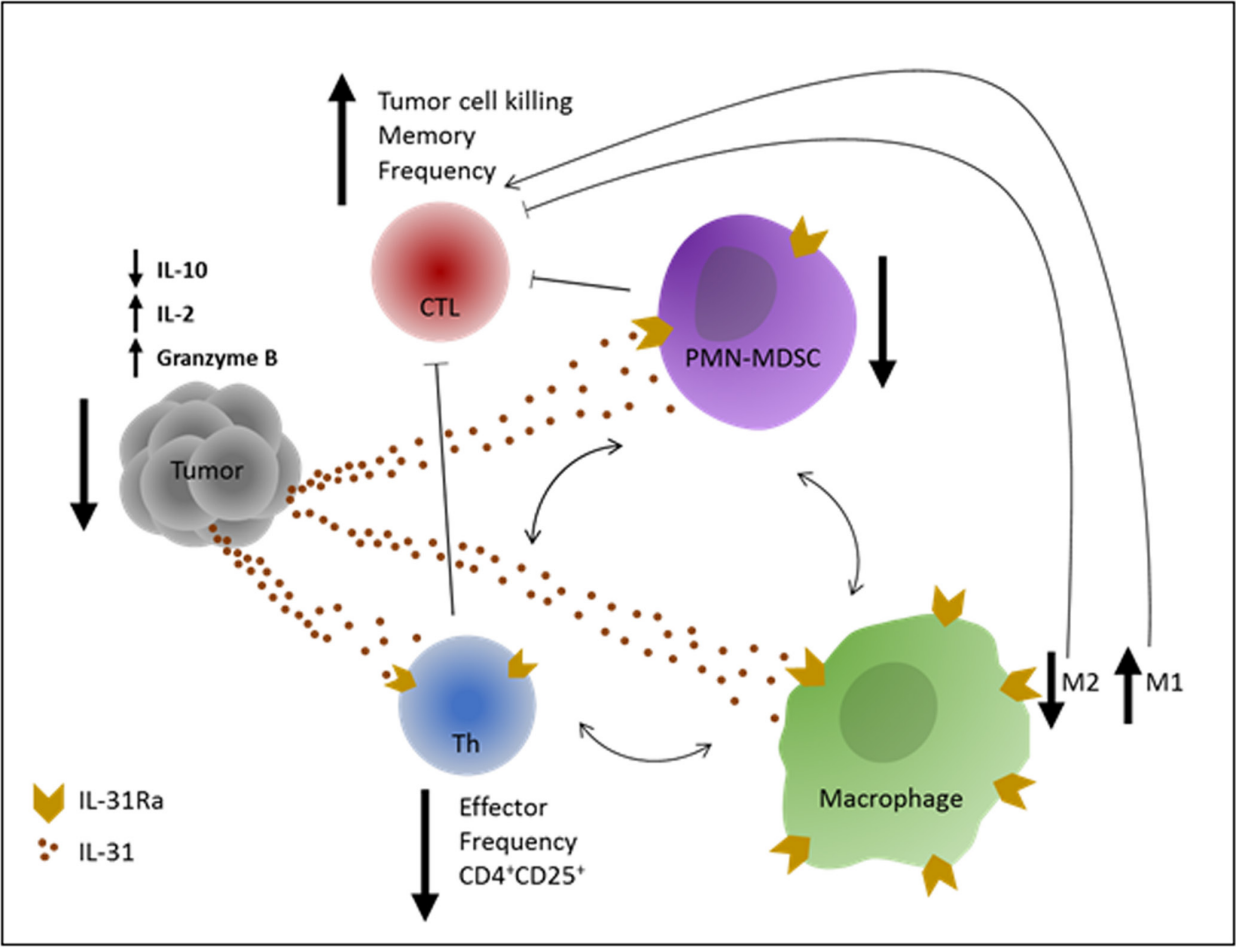
Graphical summary of IL-31 immunomodulatory effects. An illustration of the effects of IL-31 on different immune cells which contribute to antitumor immunity. IL-31 inhibits immunosuppression in the tumor by reducing PMN-MDSCs and CD4^+^CD25^+^ Th cell frequency and by promoting an M2 to M1 skewing in macrophages. In response, tumor IL-10 levels are decreased and IL-2 levels are increased. These effects indirectly promote the proliferation and antitumor activity of CTLs, leading to decreased tumor growth. In correlation with the latter, Granzyme B levels are significantly elevated in IL-31-treated tumors. Blocked thin arrows indicate suppressive effect. One-sided thin arrows indicate inducing effect. Two-sided thin arrows indicate cross-talk between immunosuppressive cell populations. CTLs, cytotoxic T lymphocytes; Th, T helper; IL, interleukin; IL-31Ra, IL-31 receptor; MDSCs, myeloid-derived suppressor cells; PMN, polymorphonuclear.

Cancer immunotherapies based on immune checkpoint inhibitors are considered game changers in the treatment of solid tumors. However, challenges still exist, including resistance to therapy in a large proportion of patients. Currently, immense research efforts are being made to discover novel immune modulators that could be used to overcome such challenges. We have previously shown that IL-31 inhibits tumor growth via antiangiogenic effects.^11^ However, since IL-31 is known to play a role in inflammation,^12^ we hypothesized that its antitumor activity could also be explained by immunomodulatory effects. Here we describe, for the first time, the immunological mechanisms by which IL-31 inhibits tumor growth. Specifically, we demonstrate that IL-31 negatively regulates immunosuppressive cell populations while indirectly increases cytotoxic T cell frequency in tumors, as summarized in figure 7.

We showed that IL-31 expression inhibited tumor growth only in immunocompetent mice but had no effect on tumor growth in immunodeficient NOD-SCID mice. While NOD-SCID mice demonstrate minor natural killer (NK) cell activity,^39^ it seems that such cells had no effect on tumor growth in IL-31-overexpressing tumors. Indeed, we found that there were no changes in NK cell population in IL-31-treated immunocompetent mice, further indicating that IL-31 may not affect NK cells. Therefore, the NOD-SCID mice experiment suggests that the major antitumor activity of IL-31 is through alterations of the immune system other than NK cells. We should note, however, that our previous study demonstrated that IL-31 inhibits both murine and human tumor growth via antiangiogenic activity and direct effect on tumor cells, which should have been observed in the NOD-SCID mouse experiment.^11^ It is plausible that the strong link found between angiogenesis and immunity^40^ in our breast cancer tumor model can explain the association between the antiangiogenic effect and immune cell alteration. To further reinforce these findings, we demonstrated that in a murine breast carcinoma model, IL-31-expressing tumors contained significantly lower numbers of immunosuppressive cell populations such as CD4^+^CD25^+^ T cells, MDSCs and M2 macrophages, but not Tregs. Accordingly, the immunosuppressive cytokine IL-10, which is mainly secreted by these populations and is known to inhibit CTL antitumor functions,^41^ was significantly decreased in IL-31-expressing tumors. Furthermore, IL-2, which drives the expansion and effector functions of CTLs as well as their differentiation into memory cells, was highly expressed in these tumors. Respectively, in IL-31-expressing tumors, a significant increase in the frequency of total CTLs and central memory CTLs followed by increased concentration of Granzyme B were found, further indicating an antitumor immunity through increased CTL activity. In an in vitro MDSC-CD8^+^ T cell co-culture system, IL-31 inhibited MDSC-mediated immunosuppression of CD8^+^ T cells, as demonstrated by increased frequency of CD8^+^CD25^+^. MDSCs have been shown before to inhibit CD25 expression in CD8^+^ cells.^42^ CD25 is part of the IL-2 receptor, and thus by inhibiting its expression MDSCs inhibit the ability of CTLs to proliferate in response to IL-2. While the frequency of CTLs with central memory phenotype increased in the co-culture system, further studies should evaluate immunological memory in an antigen-specific system. Importantly, although IL-31 did not affect the proliferation of CD8^+^ cells in our co-culture system, it seems that it allowed the induction of CD8^+^ T cell proliferation under the suppression of MDSCs. Indeed, the transwell system assay demonstrated that IL-31 inhibited the motility of PMN-MDSCs, suggesting that this is one mechanism by which IL-31 inhibits MDSC infiltration into tumors and lymphoid tissues. Taken together, our findings demonstrate the active role of IL-31 in reversing immunosuppression in the TME.

TAMs are potent immune suppressor cells that are predominant in tumors. Moreover, Th2 cytokines such as IL-4 and IL-13 are known to promote IL-31Ra expression in M2 macrophages.^34^ In vitro, we demonstrated that IL-31 enhanced the phagocytic activity of M2 macrophages and changed their mRNA expression profile toward an intermediate state between M2 and M1 macrophage function. Moreover IL-31-treated M2 macrophages secreted less IL-10 but more IL-7, which is important for proliferation and survival of CD8^+^ T cells.^43^ This fits well with our findings demonstrating decreased levels of IL-10 and increased frequencies of CD8^+^ and memory CD8^+^ cells in IL-31-expressing tumors. Thus, our results suggest that IL-31 regulates M2-TAMs by skewing their activity toward an M1-like functional state. This further promotes CD8^+^ T cell activation, facilitates phagocytosis, and suppresses the Th2 response via PTX3. We further demonstrated that IL-31 inhibited the expression of GATA3 and IL-4 Th2 factors in Th0 cells without changing the expression of Th1 factors; however, such effects were less notable under Th2-polorizing condition. Since Th2 cells are the main source of IL-31,^12^ it is plausible that IL-31 acts to regulate the type 2 immune response in Th0 cells before they become committed, similar to the regulatory effect of IL-10 in Th1 cells.^44^

The antitumorigenic activities of IL-31 highlight its potential to serve as a prognostic marker. We reported that IL-31 plasma levels inversely correlated with tumor size. We therefore investigated whether IL-31 expression correlates with good prognosis in patients with cancer as well. Our analysis of mRNA expression data sets revealed a positive correlation between survival and coexpression of IL-31Ra, IL-2 and IL-4 in breast cancer biopsies. Specifically, IL-4 has been shown to promote IL-31Ra expression in macrophages and dendritic cells in a murine model.^34 45^ While IL-31Ra expression in tumors did not correlate with increased patient overall survival, a strong correlation was found between high coexpression of IL-31Ra, IL-2 and IL-4 and increased overall survival of patients treated with neoadjuvant chemotherapy. This suggests that a causative effect between these three molecules contributes to improved overall survival in patients with breast cancer. Based on previous studies^34 45^ and our current findings, we assume that IL-4 induces the expression of IL-31Ra in tumors, which in turn promotes IL-2 expression, ultimately activating CTLs and enhancing antitumor activity. Likewise, we found that there was a correlation between IL-31Ra expression and modulation of immune cell subset, suggesting that IL-31 affects tumor immunity.

Finally, to test the therapeutic potential of IL-31 we engineered a recombinant IL-31 molecule that contains a linker region fused to an IgG heavy chain, in order to extend its half-life in circulation. Treating tumor-bearing mice with IL-31-L-IgG achieved a superior therapeutic effect in comparison with purified IL-31 delivered with osmotic pumps. Importantly, treatment with IL-31-L-IgG induced immunological changes in the TME that reflect an antitumor immune response, similar to those observed in IL-31-expressing tumors, demonstrating the potential of IL-31 to be used as a therapeutic agent.
