# Supplementary material for: IL-31 induces antitumor immunity in breast carcinoma
Source: J Immunother Cancer. 2020 Aug 24;8(2):e001010. doi: 10.1136/jitc-2020-001010 (PMC7449545; doi:10.1136/jitc-2020-001010)
Supplement: Supplementary data [file jitc-2020-001010supp001.pdf]

Supplemental Materials Online

Kan et al.

Supplemental Methods

Single cell preparation from tissue and peripheral blood

To obtain splenic single cell suspensions, mice were sacrificed, and spleens were harvested and placed in a tissue culture plate (Corning, NY, USA). The spleens were dissociated with a piston, and filtered through a 70µm cell strainer (Corning, NY, USA) to remove aggregated cells. Tumor single cell suspension was prepared as previously described [9]. To obtain bone marrow cells, tibia and femurs were removed from mice and bones were flushed using a syringe containing PBS. Peripheral blood (PB) was collected by submandibular vein puncture. The blood was collected into a tube containing 50µl 0.1M EDTA. In all methods, once single cells were obtained, red blood cells (RBCs) were lysed using RBC-lysis buffer (0.8% ammonium chloride, Alfa Aesar, Haverhill, MA, USA and 0.1M EDTA, Biological Industries, Israel).

Time of flight mass cytometry (CyTOF)

BALB/c female mice (10 weeks old) were implanted with mini-osmotic pumps (Alzet, Cupertino, CA, USA) containing 400µg of purified mouse IL-31 (PeproTech, Israel). Control mice underwent the same surgical procedure with empty pumps. After 3 weeks, mice were sacrificed and extracted spleens were prepared as single cell suspensions. The cells were acquired by CyTOF as previously described [7]. Briefly, an equal number of splenocytes were pooled per group (5 mice/group) and $3x10^6$ cells were collected from each pool for CyTOF acquisition. The cells were washed with cell staining media (PBS without Ca^{2+}/Mg^{2+}, 2% bovine serum albumin, and 0.09% Azide) and immunostained with a mix of metal tagged antibodies (listed in Table S1). Following acquisition, the cells were gated and analyzed using the viSNE algorithm on the Cytobank online platform at https://www.cytobank.org/.

Lentiviral transduction

Stable cell lines overexpressing IL-31 were generated by lentiviral transduction. The murine IL-31 and its signal peptide were cloned into an NSPI lentiviral expression vector containing a C-terminal Myc-His tag (NSPI-CMV-Myc-His; kindly provided by Prof. Gera Neufeld, Technion, Israel). Lentiviruses were generated by co-transfecting HEK-293T cells with packaging (δNRF) and envelope (VSV-G) plasmids together with NSPI-CMV-Myc-His (control) or IL-31-encoding lentiviral expression vector. Culture medium was collected 96 hours following transfection, filtered and supplemented with polybrene (8 µg/ml, final concentration). The collected medium (containing live virus) was used to infect PyMT or

EMT6 cells. Transduced cells were selected over 14 days by culturing in medium supplemented with 2 µg/ml puromycin (Sigma-Aldrich, St. Louis, MO, USA). Cells transduced with the IL-31-encoding vector are referred to as PyMT-IL-31 and EMT6-IL-31. Control cells transduced with empty vector are referred to as PyMT-ev and EMT6-ev.

CD4$^+$ Th1/Th2 polarization

Spleens from naïve C57Bl/6 mice (8-10 weeks old) were harvested and prepared as single cell suspensions. CD4$^+$ T cells were isolated by negative selection (EasySep Mouse CD4+ T Cell Isolation Kit, STEMCELL Technologies Inc.). Soluble anti-CD3 and anti-CD28 antibodies were added at a concentration of 1µg/ml to all T cell cultures. Th2 polarization was achieved by treating cultures with 10ng/ml IL-4 (PeproTech, Israel) and 10µg/ml anti-IFNγ (ThermoFisher MA, USA). Th1 polarization was achieved by treated cultures with 10ng/ml IL-12 (PeproTech, Israel) and 10µg/ml anti-IL-4 (BioLegend, San Diego, CA, USA) as previously described [6]. Th0 cells were left untreated other than the addition of activation antibodies.

Th1/Th2 cytokine quantification

To quantify cytokine secretion by Th cells in vitro, Th cells were cultured for 4 days following polarization. Cells were centrifuged and resuspended in fresh medium containing 20ng/ml phorbol 12-myristate 13-acetate (PMA) and 500ng/ml ionomycin (Sigma-Aldrich, St. Louis, MO, USA) for 18 hours. Subsequently, cells were centrifuged, and supernatant (representing CM) was collected for analysis. For tumor cytokine quantification, tumor tissue was collected, and lysates were prepared. Cytokine quantification was performed using LEGENDplex Mouse Th1/Th2 Panel (BioLegend, San Diego, CA, USA), in accordance with the manufacturer's instructions.

MDSC-CD8$^+$ T cell co-culture assay

MDSCs were isolated from PyMT tumors by positive selection. Briefly, PyMT cells were implanted in mice. When tumors reached ~800mm^3, mice were sacrificed, and tumors were extracted. Single cell suspensions of the tumors were cultured in the presence of anti-Gr1-PE antibodies (BioLegend, San Diego, CA, USA). Gr1$^+$ MDSCs were positively selected using PE selection kit (STEMCELLS, Vancouver, Canada) in accordance with the manufacturer's instructions. CD8$^+$ T cells were isolated from spleens of naïve C57Bl/6 mice (10 weeks old) after they prepared as single cell suspensions. An antibody cocktail against all splenic populations except for CD8$^+$ cells was added, in order to negatively isolate the CD8$^+$ cell population, using the manufacturer's instructions (STEMCELLS, Vancouver, Canada) in accordance with the manufacturer's instructions. Gr1$^+$ MDSCs and CD8$^+$ T cells were co-cultured in a

1:1 ratio in the presence of 1μg/ml soluble anti-CD3 and anti-CD28 antibodies (BioLegend, San Diego, CA, USA). Cell cultures were treated with 100ng/ml recombinant mouse IL-31 a dose previously determined [2] or left untreated. After 4 days, cells were immunostained with various antibodies and analyzed by flow cytometry to characterize phenotype and function. In some experiments, T cell activation was evaluated in the absence of MDSCs.

Proliferation assay

T cells ($CD4^+$ or $CD8^+$) were isolated by negative selection using a designated kit (STEMCELLS, Vancouver, Canada). Isolated cells were stained with CFSE staining reagent (BioLegend, San Diego, CA, USA) according to manufacturer's protocol. Cells were seeded at a concentration of $1x10^6$cells/ml in 96 well plates in growth medium supplemented with 1μg/ml anti-CD3 and anti-CD28 antibodies (BioLegend, San Diego, CA, USA). Cells were then treated with 100 ng/ml IL-31 for four days or left untreated. Subsequently, the cells were either fixed and stained for Ki67 according to manufacturer's protocol (BioLegend, San Diego, CA, USA), or analyzed by flow cytometry using LSRFortessa (BD Bioscience, San Jose, CA, USA). Flow cytometry data were analyzed using FlowJo v10 software (FlowJo, LLC, Ashland, OR, USA).

Bone marrow derived macrophage isolation and differentiation

Bone marrow derived macrophages (BMDMs) were isolated as previously described [10]. Briefly, bone marrow cells were flushed from the bone marrow, and subsequently cultured at a concentration of 500,000cells/ml in the presence of macrophage colony stimulating factor (MCSF, 10ng/ml, PeproTech, Israel) for seven days to induce macrophage differentiation. After seven days, medium was aspirated and replaced with fresh medium. The resulting population represents M0 macrophages. M1 and M2 macrophage skewing was carried out as previously described [5]. Briefly, M1 macrophage skewing was achieved by treating M0 macrophages with 10ng/ml IFNγ (PeproTech, Israel) and 10ng/ml LPS (Sigma-Aldrich, St. Louis, MO, USA). M2 macrophage skewing was achieved by treating M0 macrophages with 10ng/ml IL-4 (PeproTech, Israel). To analyze the secretome of macrophages in the presence of IL-31, BMDMs were treated with 10ng/ml IL-4 in the presence or absence of IL-31 (100ng/ml). After an overnight incubation, the medium was aspirated and replaced with fresh SF medium. CM was collected 16 hours later and applied to a Proteome Profiler Mouse XL Cytokine Array (R&D Systems, Minneapolis, MN, USA) according to the manufacturer's instructions.

Phagocytosis assay

Bone marrow-derived M0 macrophages were seeded in a 96-well-plate (500,000 cells/ml). The cells were either left untreated (control), treated with 10 ng/ml IL-4 to achieve M2 skewing, or treated with IL-4 (10 ng/ml) and IL-31 (100 ng/ml). In parallel, IncuCyte pHrodo Green Zymosan Bioparticles (Essen BioScience, Michigan, USA) were added to cultures (10 µg/well). Phagocytosis was evaluated and analyzed using the IncuCyte Live Cell Analysis System (Essen BioScience, Michigan, USA).

Cell deconvolution from public data

Cell deconvolution from bulk RNA of breast tumors was performed as previously described [8]. Briefly, to estimate the cell subset composition of each sample, we generated cell signatures for each cell-type from single cell gene expression data of breast cancer patients (GSE114725) in the following manner: Using the identified labeled cell-types from the study [1], we identified genes significantly different between cell-types (ANOVA, p.adj<0.05 by Benjamini-Hochberg Procedure). Per gene, we tested for differential expression the two cell types with the highest count and if significant (p<0.05) the gene was selected as a putative signature gene. Simultaneously, we calculated the entropy of each gene and identified per cell-type, the genes most specific to it. To narrow down the list of signature genes per cell-type and motivated by the understanding that the signature genes of a cell should be highly correlated within the cell type, we performed a pairwise correlation analysis between genes in the bulk gene expression data set followed by hierarchical clustering on the correlation. Per-cell type, we chose signature genes as the members of the two clusters with the highest mean correlation. Only cell-types for which at least 10 signature genes were identified were used for further analysis. Deconvolution was performed using CellMix v1.6.2 [3] and Spearman's correlation calculated between samples expressing IL-31RA (as defined above) and estimated immune cell subsets. To investigate differences in cellular milieu possibly due to IL-31, we computed a Pearson's pairwise correlation of immune cell subsets among IL-31RA expressing patients versus those whose IL-31RA was undetectable. To assess significance of correlation in IL-31RA expressing individuals we bootstrapped the IL-31RA undetectable population and tested strength of correlation obtained for equal sample size (p<0.05).

IL-31 purification

Murine IL-31 was cloned into the NSPI expression vector as previously described [2], such that it was either fused to IgG directly (NSPI-IL-31-IgG-Myc-His), or fused to a linker sequence (GGATCCGGGGGATCAGGCGGAGGTGGAGGTTCCGGTGGCGGTGGCGAATTC) followed by IgG (NSPI-IL-31-L-IgG- Myc-His). HEK-293T cells were transfected with NSPI-IL-31-IgG-Myc-His or NSPI-IL-31-L-IgG-Myc-His vectors. Stable cell lines were established by puromycin selection. To purify IL-31 fusion

Kan T, et al. J Immunother Cancer 2020; 8:e001010. doi: 10.1136/jitc-2020-001010

proteins, stable cell line cultures were serum-starved for 2 days, after which CM was collected. The medium was run through a HisTrap HP His-tag protein purification column using the ÄKTA start system (GE Healthcare, Chicago, IL, USA).

STAT3-Luciferase activation assay

To evaluate the biological activity of purified IL-31, IL-31-IgG and IL-31-L-IgG in vitro, a STAT3-luciferase assay was performed as IL-31/IL-31Ra induces STAT3 signaling pathway [4]. MEFs (4x10^5) were seeded in 60mm plates and co-transfected with an expression vector encoding murine IL-31Ra (Creative Biogene. Shirley, USA) and a reporter vector encoding luciferase controlled by the STAT3 promotor (kindly provided by Prof. Jian Li, Beckman Research Institute, City of Hope National Medical Center, CA, USA) [11]. After overnight incubation, the cells were treated with IL-31 in serum-free medium for 6 hours and then harvested. Luciferase activity was measured using the luciferase assay kit (Luciferase Reporter Assay System, Promega, CA, USA). Luminescence was quantified using a TD 20/20 luminometer (Turner Designs, Sunnyvale, CA, USA).

References

1 Azizi E, Carr AJ, Plitas G, Cornish AE, Konopacki C, Prabhakaran S *et al*. Single-Cell Map of Diverse Immune Phenotypes in the Breast Tumor Microenvironment. Cell 2018; 174: 1293-1308 e1236.

2 Davidi S, Fremder E, Kan T, Raviv Z, Timaner M, Karin N *et al*. The antiangiogenic role of the pro-inflammatory cytokine interleukin-31. Oncotarget 2017; 8: 16430-16444.

3 Gaujoux R, Seoighe C. CellMix: a comprehensive toolbox for gene expression deconvolution. Bioinformatics 2013; 29: 2211-2212.

4 Kasraie S, Niebuhr M, Baumert K, Werfel T. Functional effects of interleukin 31 in human primary keratinocytes. Allergy 2011; 66: 845-852.

5 Martinez FO, Gordon S. The M1 and M2 paradigm of macrophage activation: time for reassessment. F1000Prime Rep 2014; 6: 13.

6 Sekiya T, Yoshimura A. In Vitro Th Differentiation Protocol. Methods Mol Biol 2016; 1344: 183-191.

7 Shaked Y, Pham E, Hariharan S, Magidey K, Beyar-Katz O, Xu P *et al*. Evidence Implicating Immunological Host Effects in the Efficacy of Metronomic Low-Dose Chemotherapy. Cancer Res 2016; 76: 5983-5993.

8 Shen-Orr SS, Tibshirani R, Khatri P, Bodian DL, Staedtler F, Perry NM *et al*. Cell type-specific gene expression differences in complex tissues. Nat Methods 2010; 7: 287-289.

Kan T, *et al*. *J Immunother Cancer* 2020; 8:e001010. doi: 10.1136/jitc-2020-001010

9 Timaner M, Beyar-Katz O, Shaked Y. Analysis of the Stromal Cellular Components of the Solid Tumor Microenvironment Using Flow Cytometry. Curr Protoc Cell Biol 2016; 70: 19 18 11-19 18 12.

10 Trouplin V, Boucherit N, Gorvel L, Conti F, Mottola G, Ghigo E. Bone marrow-derived macrophage production. Journal of visualized experiments : JoVE 2013: e50966.

11 Yu CY, Wang L, Khaletskiy A, Farrar WL, Larner A, Colburn NH *et al*. STAT3 activation is required for interleukin-6 induced transformation in tumor-promotion sensitive mouse skin epithelial cells. Oncogene 2002; 21: 3949-3960.

Supplemental Tables

Supplemental Table 1 - Antibody panel used for CyTOF

#	Metal	Ab1	Clone	Supplier	Supp. cat
**1**	115In	CD45	30-F11	Biolegend	103120
**2**	141Pr	CD80	16-10A1	Biolegend	104702
**3**	142Nd	GR1	RB6-8C5	Biolegend	108402
**4**	143Nd	CD86	GL-1	Biolegend	105002
**5**	144Nd	F4/80	BM8	Biolegend	123102
**6**	145Nd	CD4	RM4-5	Biolegend	100520
**7**	146Nd	CD45R	RA3-6B2	Biolegend	103202
**8**	147Sm	Ly6c	HK1.4	Biolegend	128002
**9**	148Nd	CD138	SA011F11	Biolegend	149002
**10**	149Sm	CD8	53-6.7	Biolegend	100716
**11**	150Nd	Ly6g	1A8	Biolegend	128002
**12**	151Eu	CD206	C068C2	Biolegend	141702
**13**	152Sm	CD90	G7	Biolegend	105202
**14**	153Eu	CD14	Sa14-2	Biolegend	123302
**15**	154Sm	CD11c	N418	Biolegend	117302
**16**	155Gd	IgM	RMM-1	Biolegend	406502
**17**	156Gd	CD49b	HMα2	Biolegend	103513
**18**	157Gd	CD19	6D5	Biolegend	115502
**19**	158Gd	CD34	RAM34	BD Biosciences	553731
**20**	159Tb	CD27	LG.3A10	Biolegend	120101
**21**	160Gd	CD69	H1.2F3	Biolegend	104502
**22**	161Dy	SiglecH	551	Biolegend	129602
**23**	162Dy	TCRb	H57-597	Biolegend	109202
**24**	163Dy	CCR7	370	Biolegend	4B12
**25**	164Dy	CD28	37.51	Biolegend	102102
**26**	165Ho	CD115	AFS98	Biolegend	135502
**27**	166Er	CD133	315-2C11	Biolegend	141202
**28**	167Er	CD93	223437	R&D Systems	MAB1696
**29**	168Er	CD117	2B8	Biolegend	105802

**30**	169Tm	CD79b	HM79-12	Biolegend	132802
**31**	170Er	CD62L	MEL-14	Biolegend	104402
**32**	171Yb	CD44	IM7	Biolegend	103014
**33**	172Yb	CD43	S11	Biolegend	143202
**34**	173Yb	Sca-1	D7	Biolegend	108102
**35**	174Yb	IA-IE (MHCII)	M5/114.15.2	Biolegend	107602
**36**	175Lu	CD5	53-7.3	Biolegend	100602
**37**	176Yb	CD11b	M1/70	Biolegend	101202

A list of the full panel of metal-conjugated antibodies used for CyTOF. Antibodies were in a carrier-free format. Conjugation was performed using the MAXPAR Conjugation Kit.

Supplemental Table 2 – Definitions of immune populations according to surface markers

Population	Phenotype
T helper (Th)	$CD3^+CD8^-CD4^+$
$CD25^+$ Th cells	$CD3^+CD4^+CD25^+$
T regulatory (Treg) cells	$CD3^+CD8^-CD4^+CD25^+FOXP3^+$
Cytotoxic T cell (CTL)	$CD3^+CD8^+CD4^-$
Effector memory (Th or CTL)	$CD62L^-CD44^+$ out of T cell population
Central memory (Th or CTL)	$CD62L^+CD44^+$ out of T cell population
Macrophage	$CD45^+CD11b^+F4/80^+$
M1 Macrophage	$CD45^+CD11b^+F4/80^+CD11c^+CD206^-$
M2 Macrophage	$CD45^+CD11b^+F4/80^+CD11c^-CD206^+$
PMN-MDSC	$CD45^+CD11b^+F4/80^-$ $Ly6C^{dim}Ly6G^+$
M-MDSC	$CD45^+CD11b^+F4/80^-$ $Ly6C^{hi}Ly6G^-$

The listed surface markers were used to identify specific immune cell populations.

Supplemental Table 3 - Primers used for RT-qPCR

Gene	Forward	Reverse
Arg-1	CTCCAAGCCAAAGTCCTTAGAG	AGGAGCTGTCATTAGGGACATC
iNOS	GTTCTCAGCCCAACAATACAAGA	GTGGACGGGTCGATGTCAC
IL-10	GCTCTTACTGACTGGCATGAG	CGCAGCTCTAGGAGCATGTG
IL-7	TTCCTCCACTGATCCTTGTTCT	AGCAGCTTCCTTTGTATCATCAC
Fizz1	CCAATCCAGCTAACTATCCCTCC	ACCCAGTAGCAGTCATCCCA
PTX3	CGCAGGTTGTGAAACAGCAAT	GGGTTCCACTTTGTGCCATAAG
GATA3	CTCGGCCATTCGTACATGGAA	GGATACCTCTGCACCGTAGC
T-bet	AGCAAGGACGGCGAATGTT	GGGTGGACATATAAGCGGTTC
IFNγ	ATGAACGCTACACACTGCATC	CCATCCTTTTGCCAGTTCCTC
IL-4	GGTCTCAACCCCCAGCTAGT	GCCGATGATCTCTCTCAAGTGAT
UBE2D2 (House keeping)	ACAAGGAATTGAATGACCTGGC	CACCCTGATAGGGGCTGTC
IL31Ra	TaqMan gene ID Mm00519847_m1	
HPRT (House keeping)	TaqMan gene ID Mm00446968_m1	

mRNA expression levels of various murine genes were evaluated by RT-qPCR. Gene names and corresponding primer sequences are listed.

Supplemental Table 4 – Clinical data on breast cancer patients used in our analyses.

Age	ER status	Grade	OS [months]	Lymph node
78.6	Positive	2	12	Positive
51.46	Negative	3	12	Positive
72.49	Positive	2	97	Negative
50.21	Positive	2	74	Positive
47.13	Positive	2	99	Negative
51.23	Positive	1	88	Negative

56.1	Positive	2	74	Negative

Characteristics of breast carcinoma patients with tumoral IL-31Ra expression used for correlation and deconvolution analyses described in Figure 5. ER – estrogen receptor, OS – overall survival.

Supplemental Figures

Supplemental Figure 1 –The immunomodulatory effect of IL-31 in mice – A. Tumor-free BALB/c mice were infused with IL-31 via osmotic pumps (17µg/day) or control (n=5 mice/group). Three weeks later, mice were sacrificed and spleens were removed. Splenocytes (pooled per group) were immunostained with a 37 antibody panel as described in Table S1. Cells were acquired by CyTOF and data were analyzed by Cytobank. **A.** viSNE plot analysis of $CD4^+$ and $CD8^+$ T cell populations reveal a distinct memory phenotype elevated in IL-31-treated mice (black arrows indicate the changes in CD4, CD8 and central memory T cell populations). **B.** Macrophage population in the spleen of IL-31 treated mice displays an M1-like activation marker phenotype represented by reduced CD206 and increased CD80, CD86 and MHC II markers, as shown in histogram plot of F4/80 gated cells. **C.** Spleens and peripheral blood (PB) of IL-31-treated mice exhibit decreased levels $Gr1^+$ polymorphoneuclear cells, and $CD4^+CD25^+$ T cells (n=3 mice/group for blood or n=5 mice/group for spleens). Statistical significance was assessed by unpaired two-tailed t-test. Significant p values are shown.

Supplemental Figure 2 – IL-31-overexpressing tumors exhibit increased $CD8^+/CD4^+$ TIL ratio and decreased myeloid suppressive populations –PyMT and EMT6 stable cell lines overexpressing IL-31 (PyMT-IL-31 and EMT6-IL-31, respectively) were generated by lentiviral transduction. Control cell lines were transduced with lentivirus containing an empty vector (ev) to generate PyMT-ev and EMT6-ev. **A.** Left panel: Stable cell lines were grown to a confluency of 80%. Medium was replaced and 24 hours later, conditioned medium was collected and normalized to the number of cells. IL-31 levels in conditioned medium were measured by ELISA (n=3/group). Right panel: Proliferation of stable PyMT and EMT6 cell lines and their respective wildtype (wt) cells was measured by ATP assay over 48 hours (n=3/group). **B.** EMT6-ev or EMT6-IL-31 cells were implanted in the mammary fat pads of ten-week old female BALB/c mice (n=6/group). Tumor volume was measured. **C.** At endpoints of the experiments described in (B) and Fig. 1B, tumors were removed and peripheral blood was collected. The tumor and plasma levels of IL-31 were quantified by ELISA (n=6/group). **D.** IL-31 plasma levels in mice bearing PyMT-ev or PyMT-IL-31 were plotted against their corresponding tumor size. **E.** Mice implanted with PyMT-IL-31 tumors develop alopecia (marked by red arrows) while no such phenotype was found in mice implanted with PyMT-ev tumors. **F.** Single cells from PyMT-ev and PyMT-IL-31

tumors were immunostained for Tregs ($CD3^{+}CD8^{-}CD4^{+}CD25^{+}FOXP3^{+}$) and analyzed by flow cytometry **G.** Peripheral blood (PB) was obtained from mice bearing PyMT-IL-31 or respective control tumors. PB cells were immunophenotyped using flow cytometry (n=6 mice/group). **H-I.** Tumor single cell suspension of EMT6-ev or EMT6-IL-31 were immunostained for Th cells, CTLs, M1/M2 macrophages, and MDSCs, and analyzed by flow cytometry. **J.** Granzyme B levels were measured by ELISA in lysates of EMT6-ev and EMT6-IL-31 tumors. Statistical significance was assessed by unpaired two-tailed t-test. Correlation was assessed by Pearson correlation. Significant p values are shown.

Supplemental Figure 3 – IL-31-overexpressing tumors promote systemic anti-tumor immunity - PyMT-ev or PyMT-IL-31 cells were implanted to the mammary fat pads of 10-week old female C57Bl/6 mice. At the same time, wildtype PyMT cells were implanted in the opposing mammary fat pad in each mouse. Wildtype PyMT tumors are referred to as PyMT-wt(ev) or PyMT-wt(IL-31) according to the tumor with which it was paired. **A.** A schematic illustration of the experimental procedure is shown. **B.** PyMT-wt(ev) and PyMT-wt(IL-31) tumor growth curves are shown (n=6 mice/group). **C.** At endpoint, tumors were removed and prepared as single cell suspensions. The cells were immunostained and acquired by flow cytometry. **D**. Granzyme B levels were measured by ELISA in lysates of PyMT-wt(ev) and PyMT-wt(IL-31) tumors. **E.** IL-31 levels were measured by ELISA in tumor lysates and plasma of mice bearing PyMT tumors described in (A). **F.** PyMT-ev and PyMT-IL-31 cells were implanted into the mammary fat pad of immune-deficient NOD-SCID female mice (10-week old; n=9 mice/group). Tumor growth was plotted. Statistical significance was assessed by unpaired two-tailed t-test. Significant p values are shown.

Supplemental Figure 4 – IL-31 inhibits the expression of Th2-related cytokines in peripheral Th cells and in the tumor microenvironment - Spleens from tumor-free C57Bl/6 mice were removed and prepared as single cell suspensions. **A.** $CD4^{+}$ T cells were isolated by immunomagnetic negative selection and purity was assessed by flow cytometry. **B.** Th0 and Th2 cells were either left untreated or treated with 100 ng/ml IL-31 for 4 days. T-bet and IFNγ mRNA expression was measured by RT-qPCR (n=4 biological repeats). **C.** Ten-week old C57Bl/6 female mice were implanted with PyMT-IL31 or PyMT-ev in mammary fat pads. At endpoint, tumors were removed, and tumor lysates were prepared. The levels of various Th1/Th2 cytokines were measured (n=6 tumors/group). Statistical significance was assessed by unpaired two-tailed t-test, and no significant changes were found.

Kan T, *et al. J Immunother Cancer* 2020; 8:e001010. doi: 10.1136/jitc-2020-001010

Supplemental Figure 5 – IL-31 promotes CD8⁺ T central memory phenotype and inhibits PMN-MDSC motility – Spleens from tumor-free C57Bl/6 mice were removed and prepared as single cell suspensions. $CD8^+$ T cells were isolated by immunomagnetic negative selection. **A.** Purity of isolated $CD8^+$ T cells was evaluated by flow cytometry. **B.** $CD8^+$ T cells were treated with anti-CD3 and anti-CD28 in the presence or absence of 100ng/ml IL-31. After 18 hours the cells were assessed by flow cytometry for the T cell activation marker ($CD25^+$ T cells; n=3 biological repeats) or after 4 days for proliferation via CFSE staining. **C.** Gr1+ cells (MDSCs) were isolated from PyMT tumors. MDSC purity was evaluated by flow cytometry. **D-F.** Isolated MDSCs from PyMT tumors were co-cultured with splenic $CD8^+$ T cells in the presence or absence of 100ng/ml IL-31 for 4 days (n=4 biological repeats). The frequency of $Ki67^+$ (D) or $CD62L^-CD44^+$ effector T cell population (E) was evaluated by flow cytometry. The levels of various cytokines in conditioned medium of CD8/MDSC co-cultures were measured by multiplex analysis (F). **G**. The frequency of $CD62L^+$ or $CD44^+$ PMN-MDSC in PB of mice bearing PyMT-ev or PyMT-IL-31 tumors was evaluated by flow cytometry. Statistical significance was assessed by unpaired two-tailed t-test, and no significant changes were found.

Supplemental Figure 6 – IL-31 does not directly affect macrophage phenotype - A. BMDM purity was confirmed by flow cytometry. **B** The levels of IL-31Ra mRNA in naïve BMDMs, and in BMDMs cultured with IL-4 were measured by RT-qPCR (n=3-4 biological repeats). **C.** BMDMs were treated with IL-4 in the presence or absence of IL-31. CD11c and CD206 M1/M2 markers were evaluated by flow cytometry (n=4 biological repeats). Statistical significance was assessed by unpaired two-tailed t-test. Significant p values are shown.

Supplemental Figure 7 - IL-31Ra expression correlates with IL-2 and IL-4 expression in breast carcinoma biopsies and associated with increased survival – A. Kaplan-Meier curves of breast cancer patients who received neoadjuvant chemotherapy were plotted as a function of mRNA levels of IL31Ra, IL-2, IL-4, and IL-2+IL-4. **B-C.** Kaplan-Meier curves of all breast cancer patients were plotted as a function of mRNA levels of IL31Ra, IL-2, IL-4 and Granzyme B all together (B) or when Granzyme B or IL-31Ra is excluded (C). P values are shown. HR, hazard ratio.

Supplemental Figure 8 –In vitro and in vivo activity of mIL-31-L-IgG – A. Schematic representation of murine IL-31 fused to a linker region followed by IgG heavy chain (IL-31-L-IgG). A signal peptide (SP)

Kan T, *et al. J Immunother Cancer* 2020; 8:e001010. doi: 10.1136/jitc-2020-001010

was added at the N terminus. Myc and poly histidine tags were added at the C terminus for purification purposes. **B.** MEFs were co-transfected with expression plasmids encoding IL-31Ra and STAT3-LUC. Cells were either left untreated (control) or treated with 5µg/ml IgG, 100ng/ml recombinant mIL-31 , 2µg/ml purified mIL-31, 5µg/ml mIL-31-L-IgG or 5µg/ml mIL-31-IgG (without linker). The activities of the different IL-31 molecules were evaluated by luciferase reporter assays. **C.** IL-31-L-IgG or IgG were intraperitoneally injected twice weekly to PyMT tumor bearing mice at a dose of 100µg per injection. At endpoint, single cell suspensions from PB and spleens were prepared. The levels of $CD4^+$ T cells and MDSCs were evaluated by flow cytometry. (n=4-5 mice/group). **D.** PyMT tumors from control-IgG and IL-31-L-IgG treated mice were prepared as single cells, immunostained for Tregs ($CD3^+CD8^-CD4^+CD25^+FOXP3^+$), and analyzed by flow cytometry. Statistical significance was assessed by unpaired two-tailed T test. Significant p values are shown.

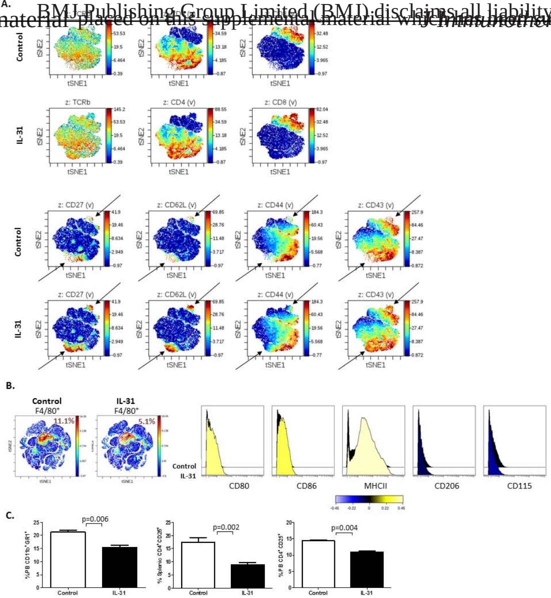
A.
Control
IL-31
tSNE1
tSNE2
z: TCRb
z: CD4 (v)
z: CD8 (v)
z: CD27 (v)
z: CD62L (v)
z: CD44 (v)
z: CD43 (v)
B.
Control F4/80+
IL-31 F4/80+
11.1%
5.1%
CD80
CD86
MHCII
CD206
CD115
C.
p=0.006
p=0.002
p=0.004
%PB CD11b+GR1+
% Splenic CD4+CD25+
%PB CD4+CD25+

Figure S2

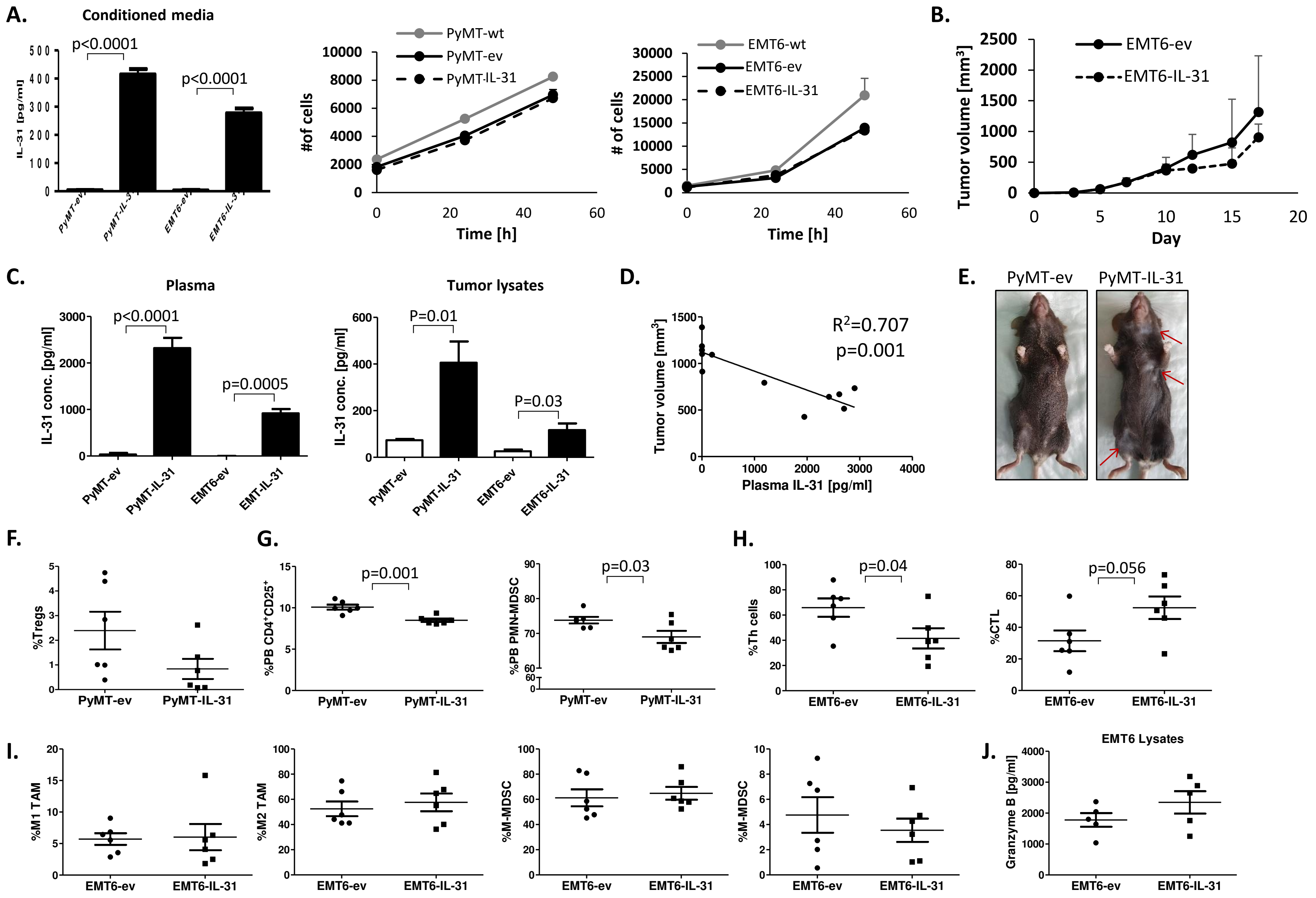
A.
Conditioned media
p<0.0001
p<0.0001
IL-31 [pg/ml]
PyMT-ev
PyMT-IL-31
EMT6-ev
EMT6-IL-31
PyMT-wt
PyMT-ev
PyMT-IL-31
#of cells
Time [h]
EMT6-wt
EMT6-ev
EMT6-IL-31
# of cells
Time [h]
B.
EMT6-ev
EMT6-IL-31
Tumor volume [mm3]
Day
C.
Plasma
p<0.0001
p=0.0005
IL-31 conc. [pg/ml]
PyMT-ev
PyMT-IL-31
EMT6-ev
EMT-IL-31
Tumor lysates
P=0.01
P=0.03
IL-31 conc. [pg/ml]
PyMT-ev
PyMT-IL-31
EMT6-ev
EMT6-IL-31
D.
R2=0.707
p=0.001
Tumor volume [mm3]
Plasma IL-31 [pg/ml]
E.
PyMT-ev
PyMT-IL-31
F.
%Tregs
PyMT-ev
PyMT-IL-31
G.
p=0.001
%PB CD4+CD25+
PyMT-ev
PyMT-IL-31
p=0.03
%PB PMN-MDSC
PyMT-ev
PyMT-IL-31
H.
p=0.04
%Th cells
EMT6-ev
EMT6-IL-31
p=0.056
%CTL
EMT6-ev
EMT6-IL-31
I.
%M1 TAM
EMT6-ev
EMT6-IL-31
%M2 TAM
EMT6-ev
EMT6-IL-31
%M-MDSC
EMT6-ev
EMT6-IL-31
%M-MDSC
EMT6-ev
EMT6-IL-31
J.
EMT6 Lysates
Granzyme B [pg/ml]
EMT6-ev
EMT6-IL-31

Figure S3

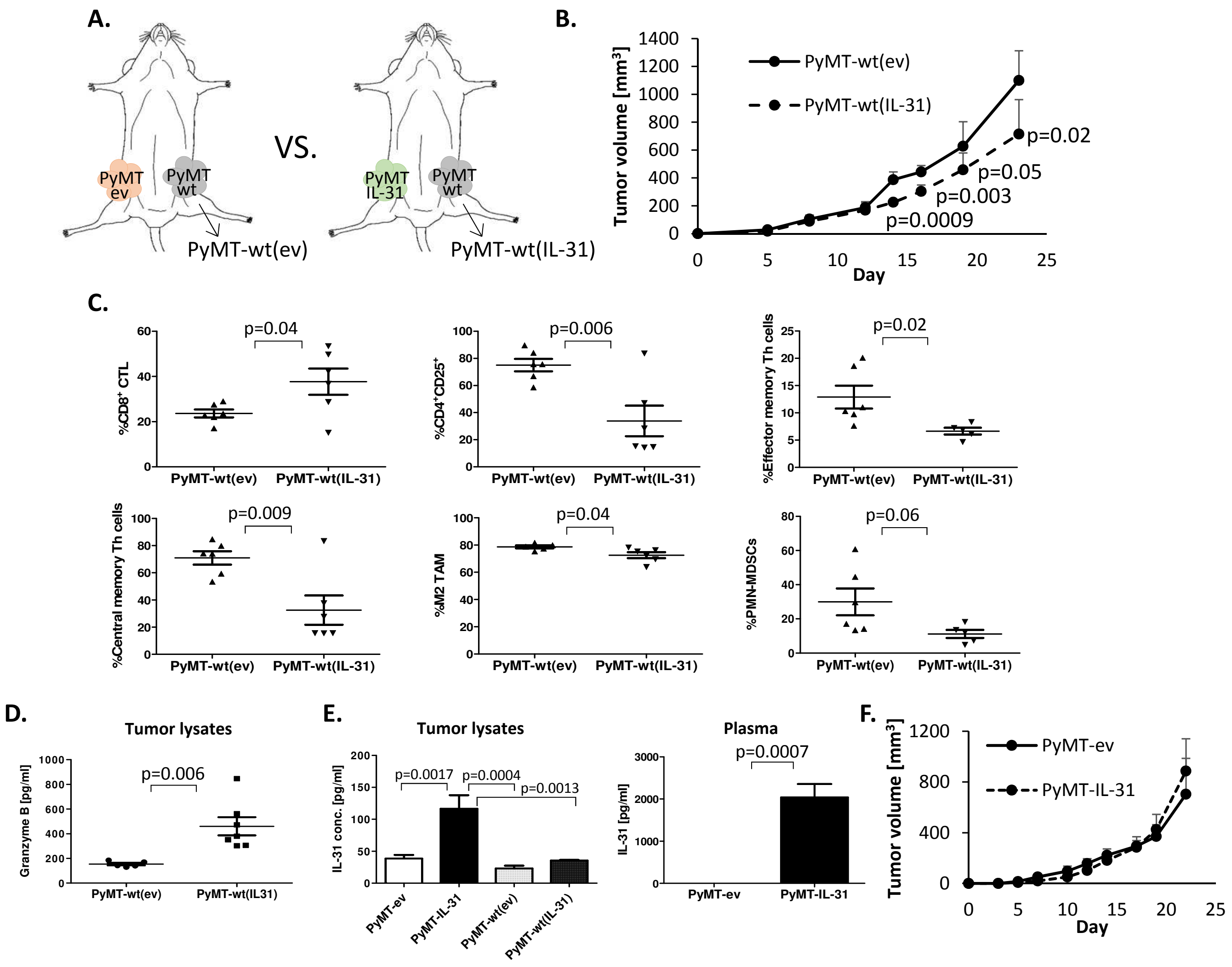
A.
PyMT ev
PyMT wt
PyMT-wt(ev)
VS.
PyMT IL-31
PyMT wt
PyMT-wt(IL-31)
B.
Tumor volume [mm3]
PyMT-wt(ev)
PyMT-wt(IL-31)
p=0.02
p=0.05
p=0.003
p=0.0009
Day
C.
%CD8+ CTL
p=0.04
%CD4+CD25+
p=0.006
%Effector memory Th cells
p=0.02
%Central memory Th cells
p=0.009
%M2 TAM
p=0.04
%PMN-MDSCs
p=0.06
PyMT-wt(ev)
PyMT-wt(IL-31)
D.
Tumor lysates
Granzyme B [pg/ml]
p=0.006
PyMT-wt(ev)
PyMT-wt(IL31)
E.
Tumor lysates
IL-31 conc. [pg/ml]
p=0.0017
p=0.0004
p=0.0013
PyMT-ev
PyMT-IL-31
PyMT-wt(ev)
PyMT-wt(IL-31)
Plasma
IL-31 [pg/ml]
p=0.0007
PyMT-ev
PyMT-IL-31
F.
Tumor volume [mm3]
PyMT-ev
PyMT-IL-31
Day

Figure S4

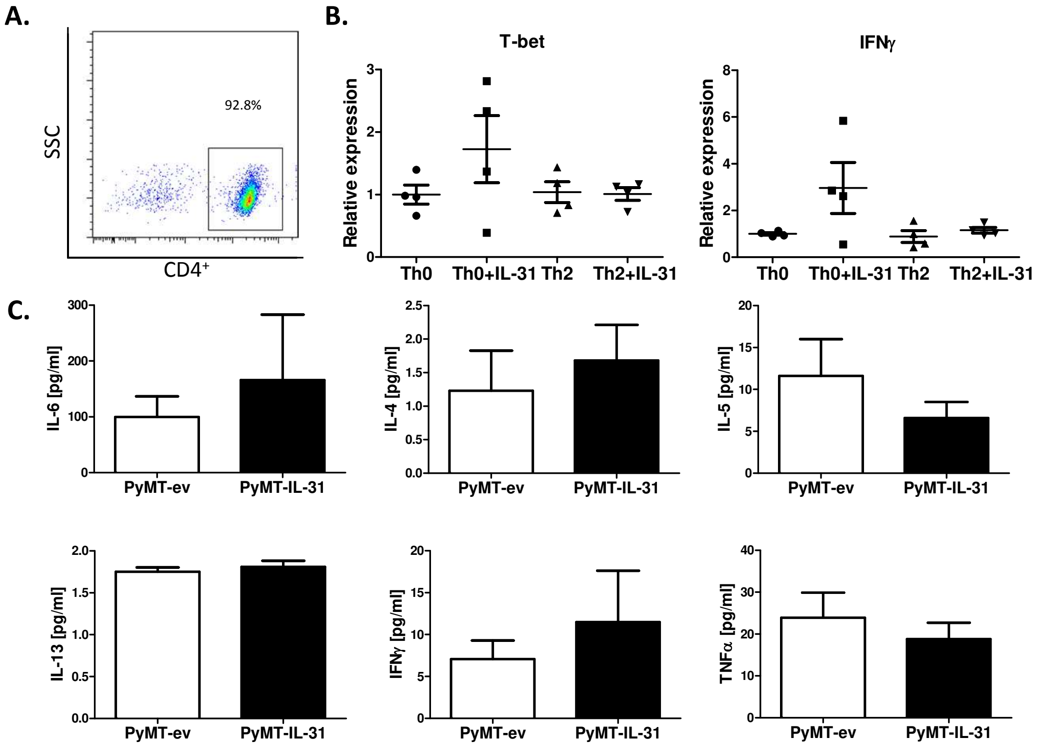
A.
SSC
CD4+
92.8%
B.
T-bet
IFNγ
Relative expression
Th0
Th0+IL-31
Th2
Th2+IL-31
C.
IL-6 [pg/ml]
IL-4 [pg/ml]
IL-5 [pg/ml]
IL-13 [pg/ml]
IFNγ [pg/ml]
TNFα [pg/ml]
PyMT-ev
PyMT-IL-31

Figure S5

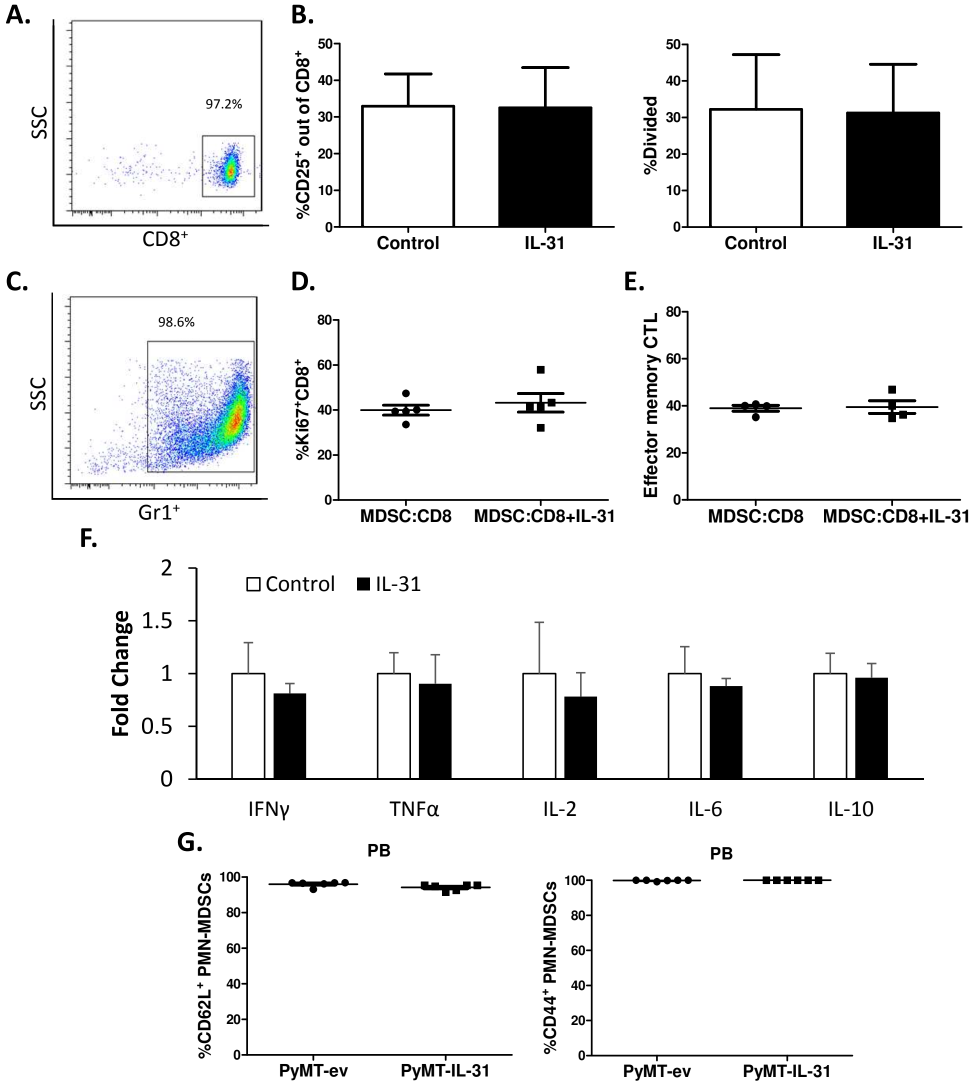
A.
SSC
CD8+
97.2%
B.
%CD25+ out of CD8+
Control
IL-31
%Divided
C.
SSC
Gr1+
98.6%
D.
%Ki67+CD8+
MDSC:CD8
MDSC:CD8+IL-31
E.
Effector memory CTL
F.
Control
IL-31
Fold Change
IFNγ
TNFα
IL-2
IL-6
IL-10
G.
PB
%CD62L+ PMN-MDSCs
%CD44+ PMN-MDSCs
PyMT-ev
PyMT-IL-31

Figure S6

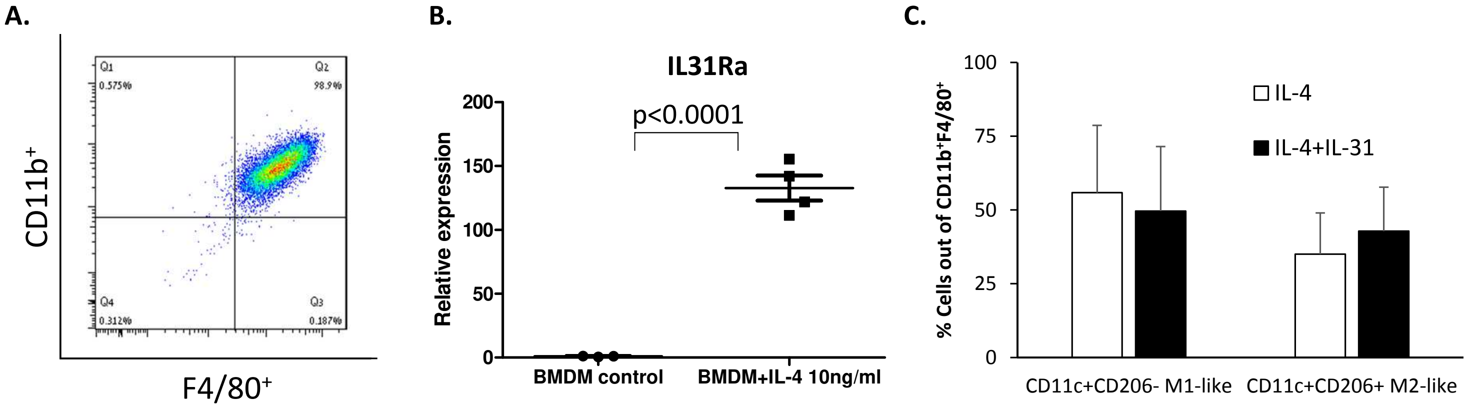
A.
CD11b+
F4/80+
Q1
0.575%
Q2
98.9%
Q4
0.312%
Q3
0.187%
B.
IL31Ra
p<0.0001
Relative expression
200
150
100
50
0
BMDM control
BMDM+IL-4 10ng/ml
C.
% Cells out of CD11b+F4/80+
100
75
50
25
0
IL-4
IL-4+IL-31
CD11c+CD206- M1-like
CD11c+CD206+ M2-like

Figure S7

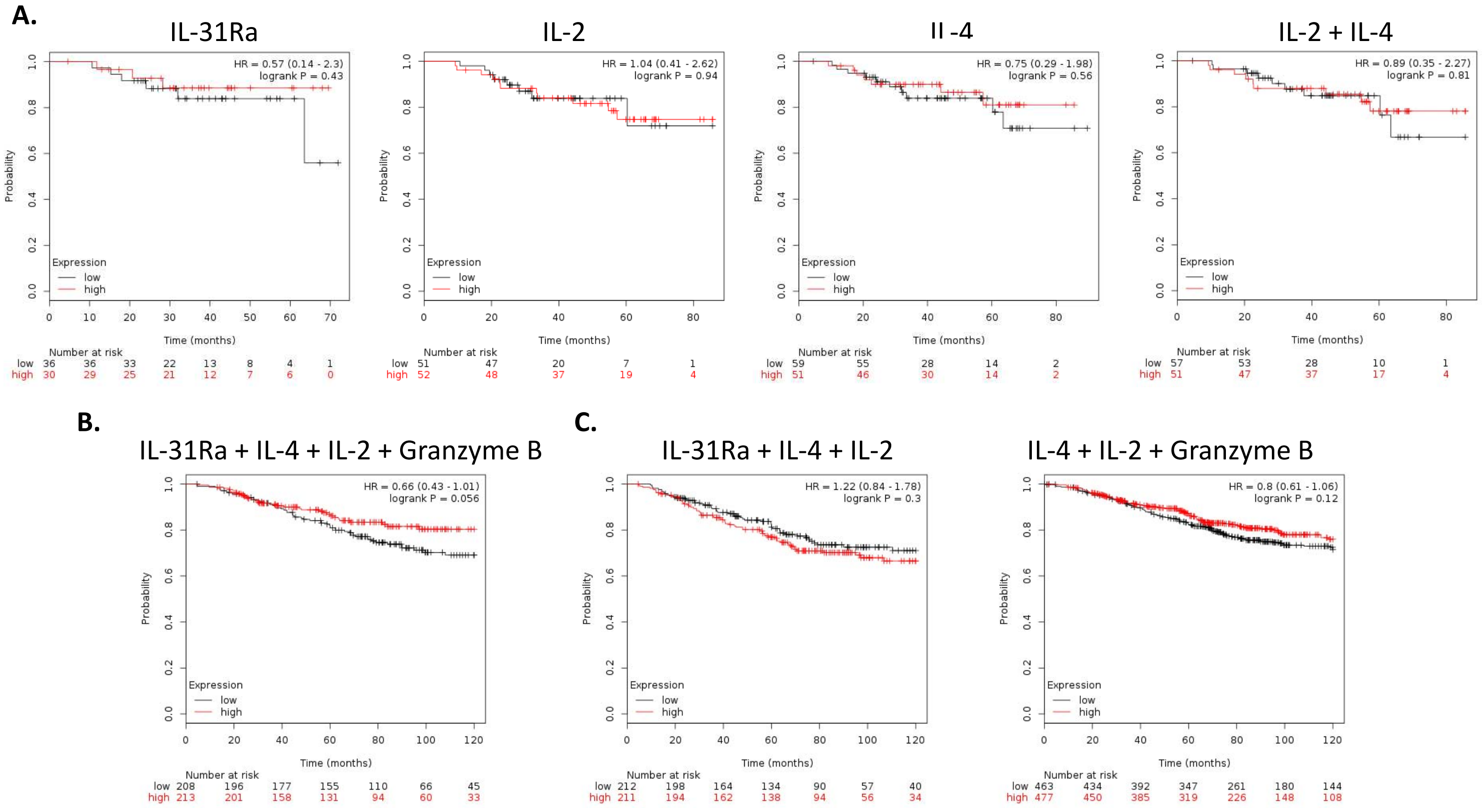
A.
IL-31Ra
HR = 0.57 (0.14 - 2.3)
logrank P = 0.43
IL-2
HR = 1.04 (0.41 - 2.62)
logrank P = 0.94
Il -4
HR = 0.75 (0.29 - 1.98)
logrank P = 0.56
IL-2 + IL-4
HR = 0.89 (0.35 - 2.27)
logrank P = 0.81
B.
IL-31Ra + IL-4 + IL-2 + Granzyme B
HR = 0.66 (0.43 - 1.01)
logrank P = 0.056
C.
IL-31Ra + IL-4 + IL-2
HR = 1.22 (0.84 - 1.78)
logrank P = 0.3
IL-4 + IL-2 + Granzyme B
HR = 0.8 (0.61 - 1.06)
logrank P = 0.12
Probability
Time (months)
Expression
low
high
Number at risk

Figure S8

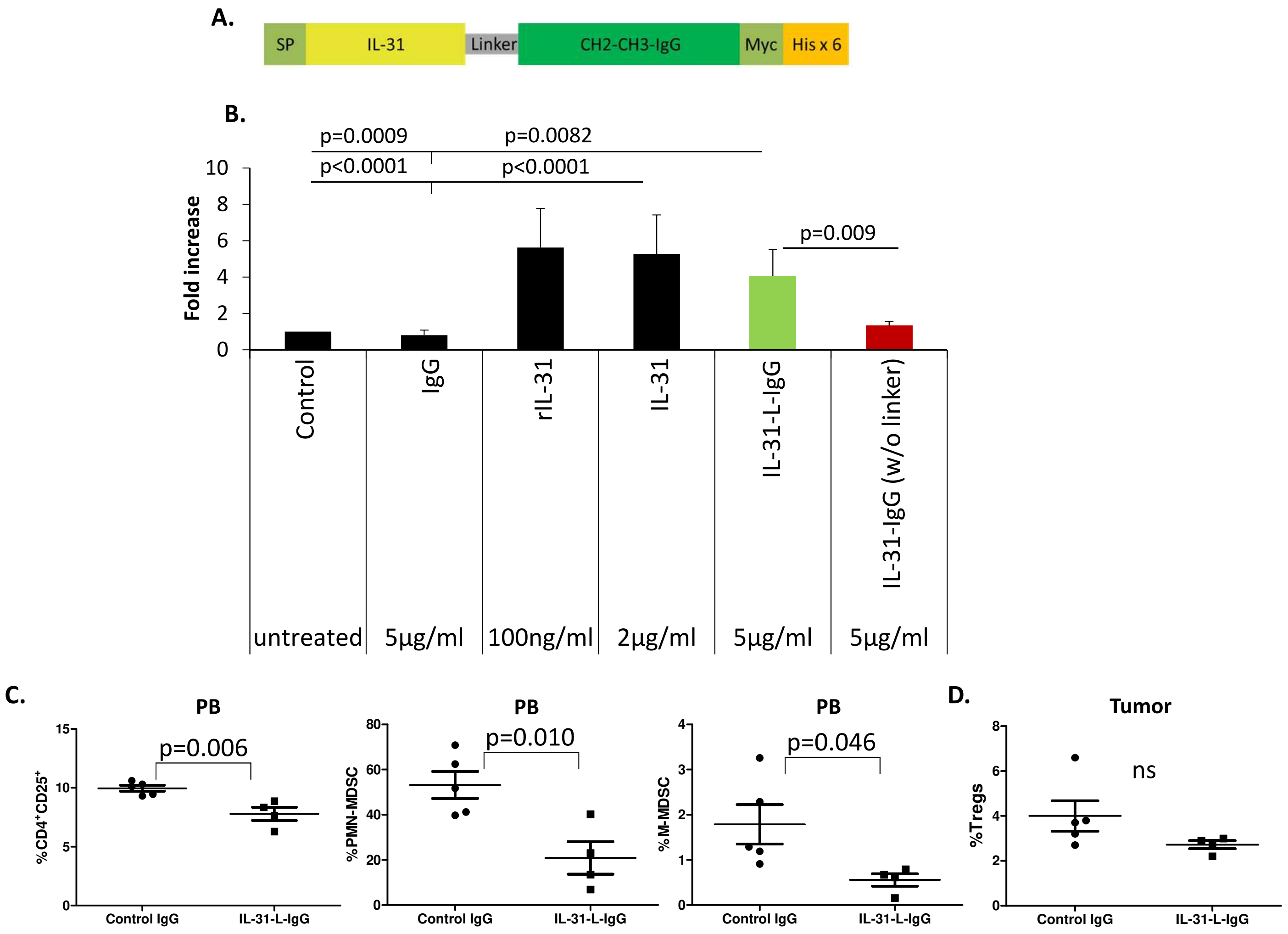
A.
SP
IL-31
Linker
CH2-CH3-IgG
Myc
His x 6
B.
p=0.0009
p=0.0082
p<0.0001
p<0.0001
p=0.009
Fold increase
Control
IgG
rIL-31
IL-31
IL-31-L-IgG
IL-31-IgG (w/o linker)
untreated
5µg/ml
100ng/ml
2µg/ml
5µg/ml
5µg/ml
C.
PB
p=0.006
%CD4+CD25+
Control IgG
IL-31-L-IgG
PB
p=0.010
%PMN-MDSC
PB
p=0.046
%M-MDSC
D.
Tumor
ns
%Tregs
